# Molecular Origins of Simultaneous Chemo‑, Enantio‑, and Substrate Selectivity in Non-Natural Photoenzymatic Radical Reactions

**DOI:** 10.1021/jacs.5c12802

**Published:** 2025-10-30

**Authors:** Felipe Curtolo, Sijia S. Dong

**Affiliations:** † Department of Chemistry and Chemical Biology, 1848 Northeastern University, Boston, Massachusetts 02115, United States; ‡ Department of Physics and Department of Chemical Engineering, Northeastern University, Boston, Massachusetts 02115, United States

## Abstract

Selective radical chemistry poses fundamental challenges for modern catalysis. Non-natural photoenzymes, most prominently flavin-dependent “ene”-reductases, have recently emerged as appealing systems to address these challenges by offering unmatched control over chemo-, enantio-, and substrate selectivity, yet their underlying photocatalytic mechanisms remain unclear. Here, we reveal the complete molecular basis of the triple selectivity control in the photoenzymatic radical reactions by the flavin-dependent “ene”-reductase *Gk*OYE-G7 through computational simulations based on multiscale multireference-quantum-mechanics/molecular-mechanics modeling and bias-exchange metadynamics. Our findings demonstrate that control emerges from reaction-level mechanisms rather than binding preferences. We discover that productive photochemistry requires a previously unknown preactivation step involving bond elongation. Stereochemical outcomes likely result from reaction barrier differences, while chemoselectivity, is controlled by crossing points between the ground and excited electronic states around the conical intersection that channel the reaction before competing pathways activate. Substrate scope follows predictable electronic-steric rules, establishing fundamental principles for engineering next-generation photoenzymes with predictable selectivity profiles.

## Graphical abstract

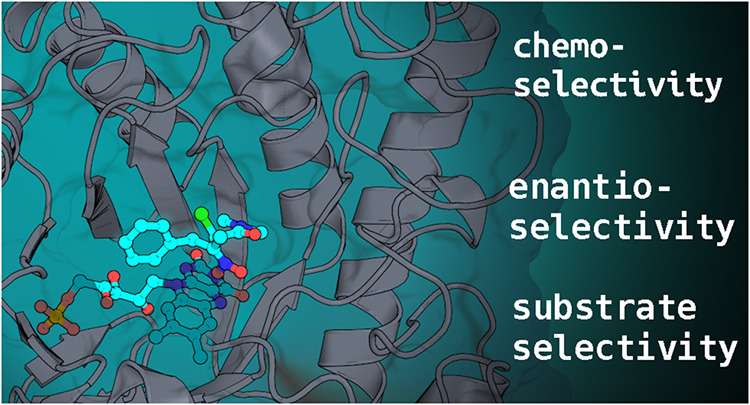

## Introduction

Controlling selectivity in radical chemistry has historically been one of the most complex challenges in catalysis.^1^ Radical intermediates are inherently reactive and prone to multiple competing pathways, making simultaneous control of chemoselectivity (which reaction occurs), enantioselectivity (which stereoisomer forms), and substrate selectivity (which substrates react) exceptionally difficult.^2^ Achieving predictable outcomes requires precise control over highly reactive intermediates, a capability that has only recently been effectively achieved by chiral photocatalysts.^3^

Non-natural photoenzymes, which are characterized by their ability to make use of a cofactor to promote charge transfer upon photoexcitation and enable non-native catalytic reactions, have emerged as powerful tools for such control and have gained increasing interest in both academia and industry.^4–12^ Such enzymes do not rely on light-driven chemistry to function in nature, but can be repurposed and engineered to be photocatalysts. Prominent examples of non-natural photoenzymes are flavin-dependent “ene”-reductases (EREDs),^13^ which involve the flavin mononucleotide (FMN) cofactor in the photochemistry. It is believed that, upon light absorption, charge transfer occurs between the flavin cofactor and the substrate, generating carbon-centered radicals within the enzyme’s active site.^14,15^ These radicals can undergo cyclizations,^4,5^ C–C couplings,^6,7^ or radical additions^8,9^ before typically terminating through hydrogen-atom transfer from the flavin cofactor.^4–8^ Despite using achiral substrates, these systems routinely achieve more than 90% enantiomeric excess through mechanisms involving precise spatial control of both radical formation and termination.^15,16^

However, the molecular basis for simultaneous control of all three selectivity types remains poorly understood. While individual aspects have been studied, particularly enantioselectivity,^17,18^ the underlying mechanisms by which photoenzymes achieve chemoselectivity, enantioselectivity, and substrate selectivity together have not been systematically characterized. This lack of mechanistic understanding prevents rational design of photoenzymes with tailored selectivity profiles.

The flavin-dependent photoenzyme *Gk*OYE-G7 exemplifies both the potential and the mystery of photoenzyme selectivity control.^9^ This enzyme catalyzes redox-neutral C-alkylation of nitroalkanes with α-halo carbonyl compounds (Figure 1a), displaying distinct chemoselectivity compared to related photoenzymes that favor cross-electrophile coupling (XEC).^7^ It achieves high enantioselectivity through a mechanism that does not rely on FMN positioning for hydrogen-atom transfer during termination, yet accepts diverse nitroalkanes while rejecting many α-halo carbonyl variants for unknown reasons^9^ (Figure 1b).

**Figure 1:**
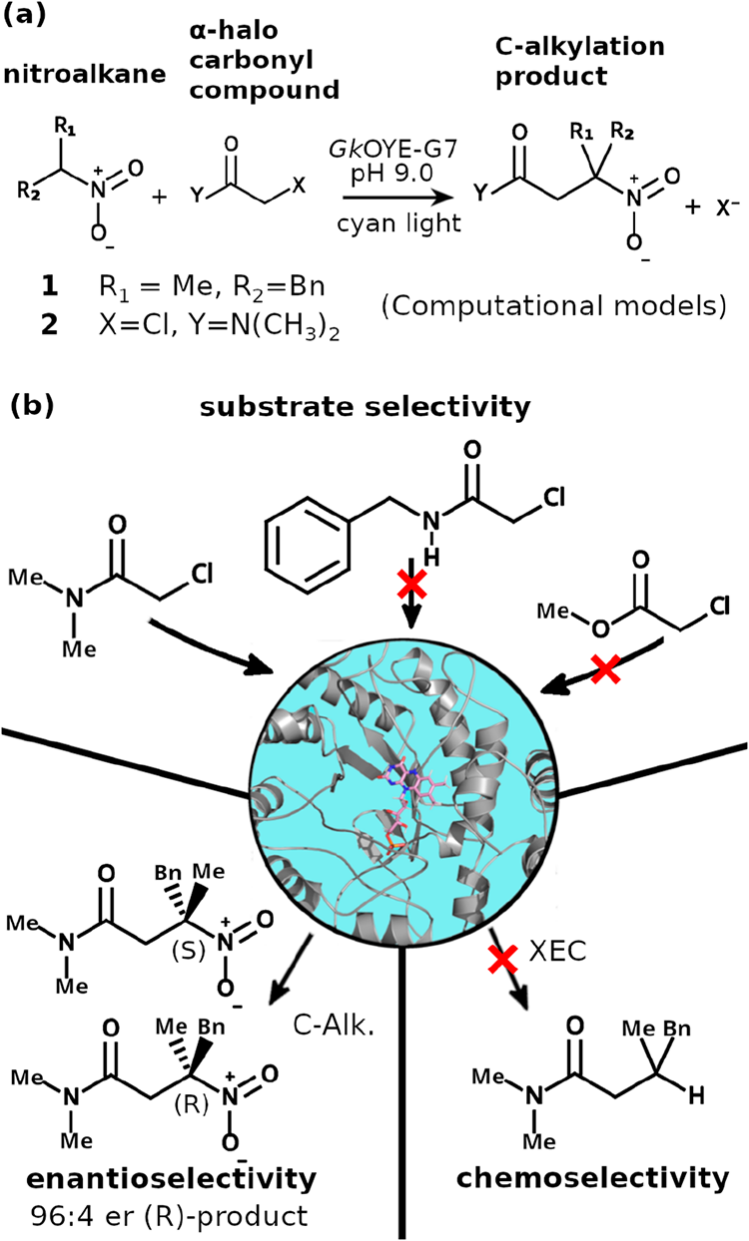
Triple selectivity control in the photoenzyme *Gk*OYE-G7. (a) General reaction scheme for the C-alkylation of nitroalkanes with α-halo carbonyl compounds catalyzed by *Gk*OYE-G7 under cyan light irradiation. Computational models employed substrates **1** (R_1_ = Me, R_2_ = Bn) and **2** (X = Cl, Y = N­(CH_3_)_2_). (b) Overview of the three selectivity challenges addressed in this work. *Substrate selectivity*: The enzyme accepts the 2-chloro-*N,N*-dimethylacetamide substrate (left) but rejects some amide and ester variants (top, red X’s). *Chemoselectivity*: Among possible radical pathways, *Gk*OYE-G7 favors C-alkylation over cross-electrophile coupling (XEC, red X). *Enantioselectivity:* The reaction produces predominantly the (R)-configured product with high selectivity (96:4 er).^9^

Although the original experimental characterization of *Gk*OYE-G7 proposed that the reaction proceeds through charge-transfer complex formation between the reduced FMN and the α-halo substrate followed by radical generation,^9^ the molecular basis for selectivity control remained unclear. Moreover, no experimental structure exists showing substrate organization within the active site, leaving the structural and mechanistic determinants of selectivity unexplored.

Here, we reveal the molecular origins of triple selectivity control in *Gk*OYE-G7 through integrated computational simulations. We first characterized the structural organization of enzyme–substrate complexes using bias-exchange metadynamics.^19^ These simulations, combined with multireference quantum mechanics/molecular mechanics (QM/MM) calculations,^20,21^ demonstrated that enantioselectivity likely arises from lower reaction barriers for the productive stereochemical pathway, chemoselectivity is influenced by early appearing conical intersections with high driving force that commit the reaction before alternative pathways become accessible, and substrate selectivity operates via balanced electronic and steric requirements that control access to productive preactivation states. These findings establish design principles for selective photoenzymes and demonstrate the first systematic control of all three selectivity types in a photoenzyme.

## Results and Discussion

### Substrate Binding Preferences Alone Cannot Explain Enantioselectivity

Enantioselectivity in enzyme catalysis is traditionally attributed to the classical “lock-and-key” model, where preferential substrate binding in productive conformations determines stereochemical outcomes.^22^ To test whether this mechanism explains *Gk*OYE-G7’s remarkable 96:4 enantioselectivity in the C-alkylation of nitroalkane **1** and α-chloroamide **2**, we performed enhanced sampling molecular dynamics simulations (MD) with bias-exchange metadynamics using collective variables that track the position of the substrates relative to the active site (8 biased replicas × 500 ns, triplicate, 12 μs total) at 300 K and 1 bar to explore substrate binding preferences (see Computational Methods and Supporting Information (SI) Sections S1.1 and S2.2 for detailed collective variables definition and convergence analysis).

Unexpectedly, both substrates exhibit weak, nonspecific binding that contradicts traditional selectivity models. The binding free energy profile of **1** in its deprotonated nitronate form (the reactive anionic species in pH 9 ^23^) reveals a wide, relatively shallow binding well, with the active site region showing the lowest binding free energy of −6.8 kcal/mol (Figure 2a). However, binding in the active site region is not significantly more stabilizing than binding in adjacent regions as shown by the relatively flat energy profile at greater substrate distances, contrasting sharply with traditional enzymes that rely on complementary “lock-and-key” interactions. We observed multiple metastable states with similar energies throughout the 17–30 Å region, allowing a variety of possible binding modes for both **1** and **2**. Similar binding flexibility has been reported in other photoenzyme active sites, suggesting that conformational heterogeneity may be a common feature in these systems.^5,18^

**Figure 2:**
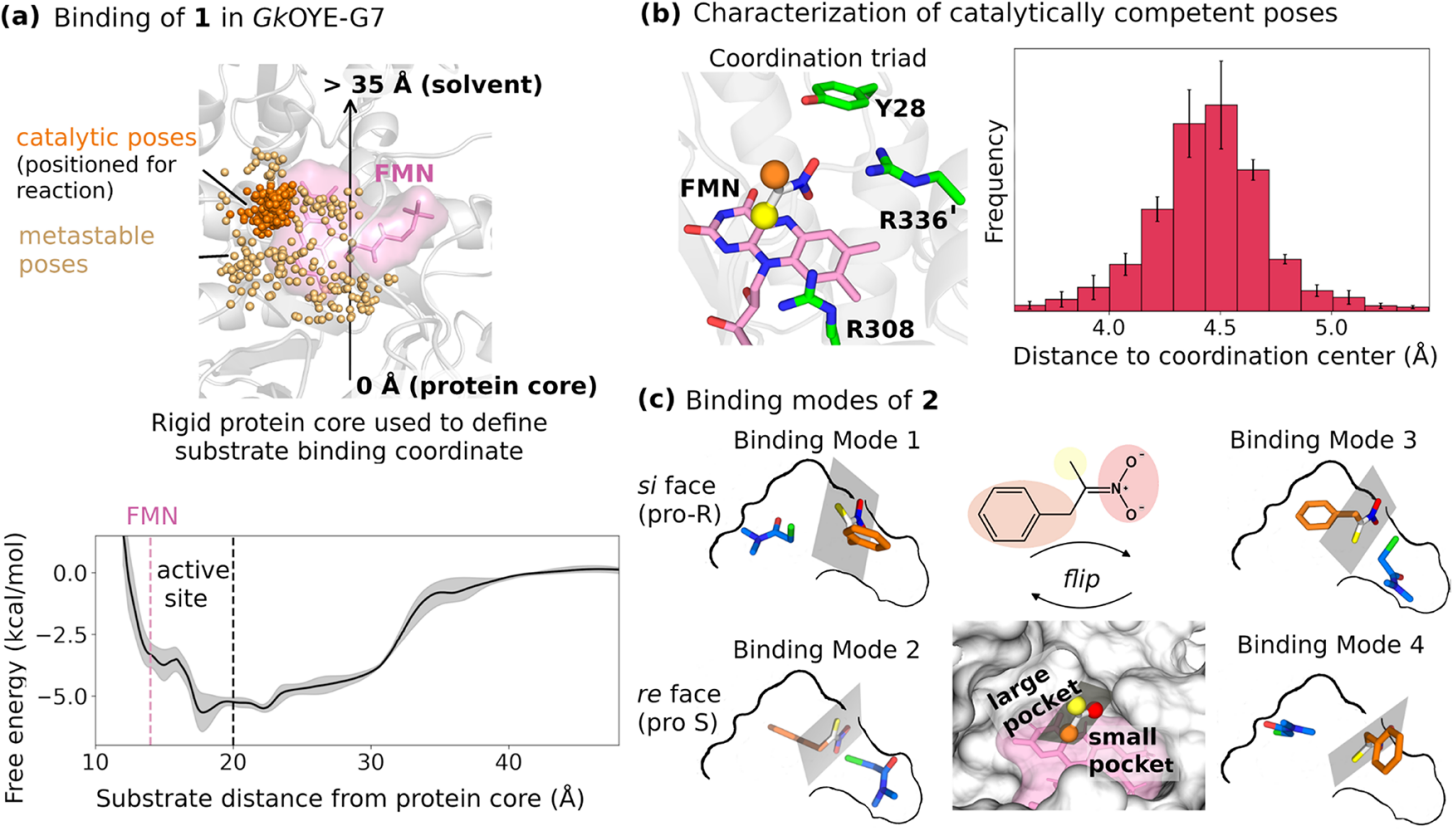
Substrate binding analysis reveals insufficient selectivity for enantiocontrol. (a) Binding free energy landscape of nitronate **1** in *Gk*OYE-G7 along a coordinate that tracks the substrate’s distance from the active site (one of the collective variables used; see Computational Methods for details). Top: Representative binding poses showing catalytic conformations (orange) and metastable states (beige) throughout the binding landscape (definition in SI Section S2.4). Bottom: Free energy profile reveals shallow binding with multiple accessible states. Pink dashed line indicates FMN position; black dashed lines denotes active site boundary; shaded region indicates standard errors over *n* = 3 independent simulations. (b) Characterization of catalytically competent poses. Left: Coordination triad (Y28, R308, R336’) that orients the nitronate group of **1** (shown in stick representation with FMN in pink). Right: Distance distribution histogram showing consistent nitronate positioning relative to coordination center; error bars represent standard error. (c) Binding modes of α-chloroamide **2** (blue) in catalytically competent poses. Four distinct binding configurations are observed relative to the nitronate plane: Modes 1 and 3 approach the *si* face (pro-R) and modes 2 and 4 approach the *re* face (pro-S). Benzyl group is shown in orange and methyl group in yellow to clarify stereochemical assignments. Center: Cross-section of the active site showing large and small binding pockets created by nitronate coordination. The multiple accessible binding modes for **2** demonstrate that binding preferences alone cannot account for the observed 96:4 enantioselectivity.

To validate this observation, we compared our simulations to experimental binding affinities in similar enzymes. Our calculated standard binding free energy (Δ*G*_bind_^°^ = 2.92 ± 0.35 kcal/mol) corresponds to a *K*_D_ of 7.2 mM, indicating low binding affinity. This is not atypical for “ene”-reductases, which exhibit *K*_M_ values in the mM range for their own natural substrates.^24^ We attribute the low affinity to high flexibility of loops surrounding the active site (RMSF analysis in Supporting Information section S2.3), a ubiquitous feature in these enzymes^25^ that represents an attractive target for engineering.^26^

Despite multiple binding possibilities, reactions proceed only when substrates and FMN are organized to form an electron donor–acceptor complex (EDA).^4,14^ To identify catalytically relevant binding modes that could facilitate such EDA formation, we filtered bound conformations to select only those with both substrates positioned near FMN in geometries with π-stacking electronic interactions (detailed filtering metrics in Supporting Information Section S2.4). Among these catalytically competent poses, we observed that while **1** can flip around the nitronate plane, the nitro group itself remains rigid and consistently orients toward a triad of residues (Y28, R308, and R336’ from the adjacent chain) that forms a coordination center (Figure 2b). The most likely distance between the centers of mass of the nitro group and of the triad side chains is 4.5 Å, with at least one hydrogen-bond between them.

Tyrosine residues have p*K*_a_ values near 9–10 in aqueous solution, suggesting that Y28 could potentially be deprotonated at the experimental pH of 9.0 and thus unable to engage in hydrogen bonding with **1**. In contrast, the arginine residues R308 and R336’ are likely to remain protonated due to their naturally high p*K*_a_ values (>12 in solution). Since our binding simulations assumed that Y28 is protonated, we performed constant pH molecular dynamics simulations (CpHMD) on three catalytically competent geometries with Y28 as a titratable residue to validate this assignment. The CpHMD simulations (100 ns each) revealed that Y28 remained protonated for more than 99% of the simulation time (Supporting Information Section S2.6), confirming its ability to serve as a hydrogen-bond donor to the nitro group of substrate **1** and supporting the coordination triad model.

Because the triad residues exhibit low flexibility (average RMSF = 1.6 ± 0.4 Å), the nitronate position in catalytically competent poses remains relatively constant, effectively dividing the active site into two distinct binding pockets (Figure 2c). We calculated average pocket volumes of 110 Å^3^ (small pocket) and 160 Å^3^ (large pocket), using KVFinder.^27^ These pockets readily accommodate different R_1_ and R_2_ substituents in **1**, explaining why *Gk*OYE-G7 accepts diverse nitroalkanes with varying sizes.

In contrast, **2** exhibits less constrained binding behavior. Geometric classification of catalytically competent poses reveals four possible binding modes: two pro-R configurations with **2** positioned at the *si* face of the nitronate plane, and two pro-S configurations at the opposite *re* face (Figure 2c). As a small molecule molecular volume = 111 Å^3^, calculated from the van der Waals radii, **2** shows no clear binding preferences and even weaker binding affinity than **1**, with Δ*G*_bind_^°^ = 0.5 kcal/mol (binding free energy landscape in Supporting Information Section S2.5).

Despite the relatively constant orientation of **1**, the adjacent binding pockets are sufficiently large to accommodate **2** in multiple configurations, making it unclear how substrate binding alone could direct stereoselective bond formation with 96:4 enantiopreference.^9^ While binding preferences may exist among the four possible modes, our extensive simulations (12 μs total) could not identify a clearly favored **2** binding mode with reasonable statistical confidence. This finding explains why photoenzymes can accommodate diverse substrate classes with different sizes, a key feature for synthetic applications, but strongly suggests that binding preferences alone cannot account for the high degree of enantioselectivity observed.

### Reaction Mechanism Reveals Origins of Triple Selectivity

The absence of binding-based enantioselection in *Gk*OYE-G7 indicates that stereochemical control must emerge from reaction-dependent mechanisms. This finding, likely general to flexible photoenzymes, motivated detailed investigation of the photochemical reaction mechanism underlying both C-alkylation and cross-electrophile coupling (XEC) between **1** and **2**. Because the reaction involves radical formation in a highly heterogeneous enzyme environment, we employed high-level multireference QM/MM calculations, using multireference methods in the QM region to accurately model radical photochemistry and using electrostatic embedding to incorporate the protein environment’s effects. To our knowledge, this represents the first application of multireference QM/MM calculations to study the complete reaction pathway in non-natural photoenzyme catalysis. Key reaction intermediates possess substantial diradical character, which conventional single-reference methods like time-dependent density functional theory (TD-DFT) fail to describe accurately,^21,28^ making our multireference treatment essential for reliable energetics (see model validation in Supporting Information Section S2.7).

Building on earlier hypotheses,^9^ our calculations reveal that cyan light absorption forms a charge-transfer (CT) state between FMN and substrate **2** (geometry **I**, Figure 3a), triggering chloride dissociation to generate the radical intermediate **II**. C–C bond formation then begins (geometry **III**), leading to a critical bifurcation where the reaction can proceed downhill through a conical intersection representing back electron transfer to form the C-alkylation product (geometry **IV**), or alternatively follow the XEC pathway without back electron transfer.

**Figure 3:**
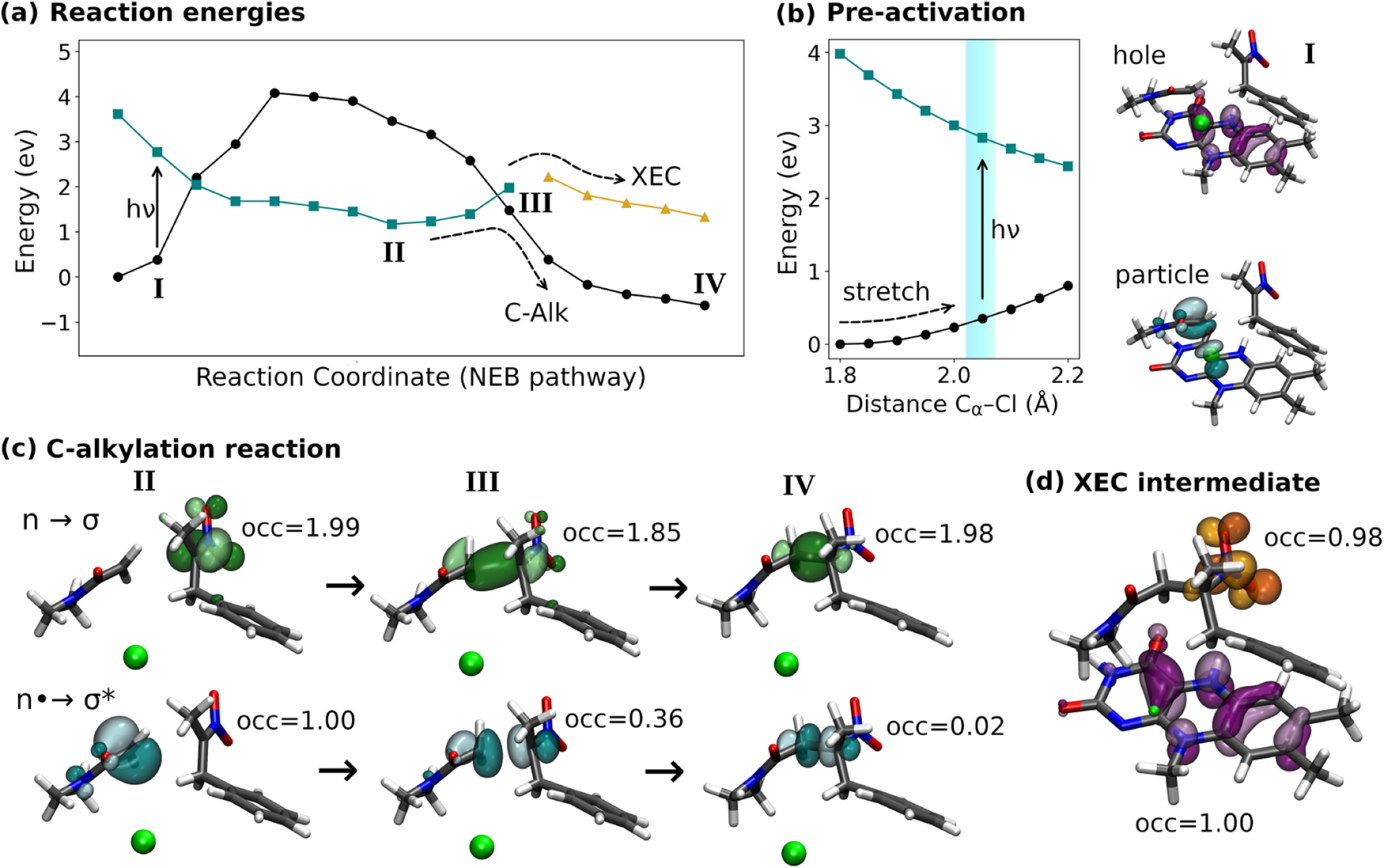
Multireference QM/MM calculations reveal the photochemical mechanism and selectivity control points. (a) Reaction energy profiles showing closed-shell states (black circles), charge-transfer state (cyan squares), and XEC nitro-radical anion (orange triangles). Roman numerals indicate key geometries along the NEB optimized reaction pathway: **I** (preactivated geometry that absorbs cyan light), **II** (radical anion intermediate), **III** (crossing point), and **IV** (C-alkylation product). (b) Preactivation mechanism showing excitation energy dependence on C_α_–Cl bond length. Productive charge-transfer requires bond elongation to 2.0–2.1 Å (cyan region). Molecular structures show hole and particle natural transition orbitals illustrating the charge-transfer character. (c) Orbital evolution during C-alkylation showing transformation of nonbonding orbitals into C–C bonding/antibonding orbitals. Occupation numbers (occ) track electron redistribution as the reaction progresses from radical anion **II** to product **IV**. Green spheres represent dissociated chloride ions. (d) XEC nitro-radical anion state showing nitro π* orbital and flavin radical orbital with 1 electron each. This state is a reaction intermediate of XEC.

#### Preactivation Mechanism Enables Productive Photochemistry

We found that productive photochemistry requires an important preactivation step involving C_α_–Cl bond elongation in **2**. Effective CT formation with cyan light requires prior stretching of the C_α_–Cl bond from about 1.8 Å to 2.0–2.1 Å, which lowers the σ* orbital energy and enables productive photon absorption at 2.5 eV (cyan/blue light) rather than the 3.6 eV required at equilibrium geometry according to our calculations (Figure 3b).

This preactivation mechanism likely represents a general feature of photoenzyme catalysis. The 8 kcal/mol (0.35 eV) energy required for C_α_–Cl bond elongation corresponds to approximately 13 kT at 300 K, placing such conformations on the microsecond time scale, rare but accessible thermally. This process competes with nonproductive local FMN excitation that rapidly decays to the ground-state,^29^ creating selectivity challenges for the productive photochemistry. Capone et al. observed related orbital alignment requirements in photoenzyme GluER-G6 and noted predominant nonproductive excitation over CT formation.^30^ However, their 200 ns MD simulations likely sampled primarily equilibrium conformations, potentially missing the stretched bond geometries we identify as critical for productive photochemistry with QM/MM reaction path calculations.

While α-chloroamide **2** participation in the CT formation is evident from the particle/hole orbitals (Figure 3b), we investigated whether substrate **1** contributes electronically to this process or serves solely a steric role in organizing the active site. Deletion of **1** from the binding pocket while maintaining geometry revealed identical cyan light excitation (difference of only 0.04 eV) with unchanged natural transition orbitals, demonstrating that **1** does not electronically modulate the charge-transfer process. Instead, our calculations indicate that **1** plays a structural organization role: it coordinates with the binding triad to create binding pockets, while **2**’s electronic properties control charge-transfer efficiency. This distinct role explains the observed substrate selectivity patterns: *Gk*OYE-G7 exhibits broad tolerance for nitroalkane variants (which should maintain similar binding through triad coordination) but stringent selectivity toward α-halo carbonyl compounds (whose electronic properties impact photochemical reactivity).^9^

#### Enantioselectivity Arises from Differential Binding Mode Barriers

To understand the reaction and validate our computational approach, we analyzed orbital evolution during C-alkylation (Figure 3c). At intermediate **II**, the nitronate **1** possesses a doubly occupied nonbonding orbital (*n*), while **2** exhibits a singly occupied orbital formed after heterolytic chloride dissociation and electron transfer from FMN (*n* ^•^). As the substrates approach, these orbitals reshape into σ and σ* orbitals of the forming C–C bond, ultimately sharing two electrons with the excess electron returning to regenerate FMN.

Due to the state crossing observed in the reaction profile, nonadiabatic transitions likely play a role in the kinetics of product formation. To quantify these effects, we calculated nonadiabatic couplings (NAC) between the ground and excited states along the reaction pathway at the SA-CASSCF level.^31^ At geometry **III**, the energy gap becomes minimal and the derivative coupling peaks (Figure 4a), indicating efficient nonadiabatic decay to the ground state toward the product.

**Figure 4:**
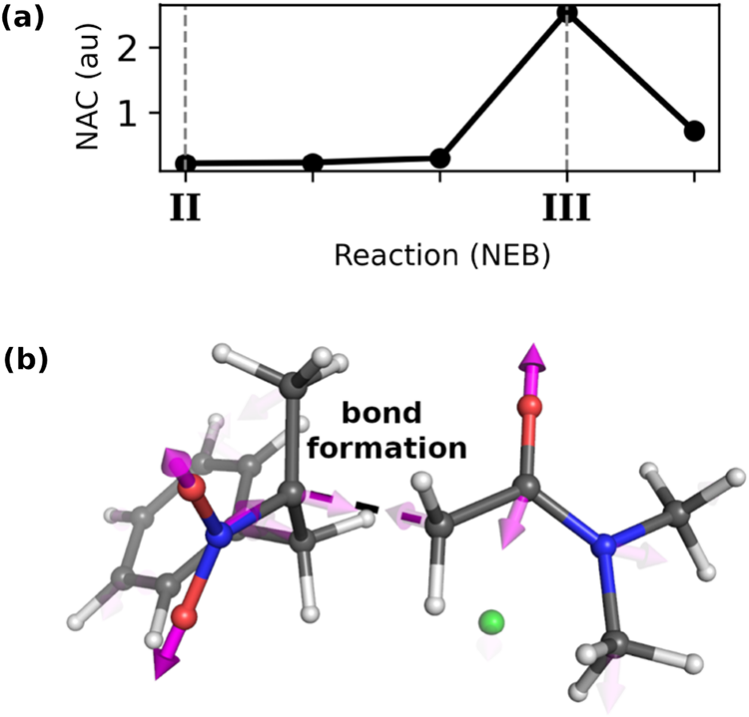
(a) Root-mean-square (RMS) of the nonadiabatic coupling vector (NAC) between ground and excited states as a function of reaction coordinate, showing peak coupling at geometry **III**. (b) Nonadiabatic coupling vector at geometry **III**, with vector opacity indicating coupling strength. Strong coupling components align with C–C bond formation (indicated by dashed line) and nitro, carbonyl vibrations, while most atoms show negligible coupling (transparent vectors).

Our analysis reveals that strong coupling occurs specifically at the geometry **III**, while surrounding geometries show negligible coupling values. This indicates that nonadiabatic transitions can only occur efficiently when the system reaches the crossing region near **III**. Since geometry **III** lies energetically uphill from intermediate **II**, the system must first overcome the energy difference between these points to access the region where nonadiabatic transitions become feasible. Therefore, we used the energy required to reach geometry **III** as an approximate measure of the barrier for accessing the product-forming pathway. Even though **III** is not an optimized conical intersection, this approach should provide a reasonable basis for comparing binding modes and correlating with stereochemical outcomes.

We calculated these barriers for three randomly selected geometries from three binding modes identified in Figure 2c. We omitted mode 4 due to computational expense and focused on modes representative of both stereochemical outcomes: modes 1 and 3 (pro-R conformations) and mode 2 (pro-S conformation) (see Supporting Information Section S2.8 for complete reaction paths).

These calculations suggest differences among binding modes that may contribute to enantioselectivity. For the geometries tested, binding mode 1 exhibits the lowest barrier (7.8 ± 3.5 kcal/mol), while both binding mode 3 (also pro-R) and binding mode 2 (pro-S) show higher barriers (13.2 ± 2.5 and 13.7 ± 0.5 kcal/mol, respectively). The 5–6 kcal/mol energetic advantage of binding mode 1 suggests enantioselectivity might be driven by favorable reaction kinetics, rather than through preferential binding or simply pro-R positioning. While the computational expense of multireference QM/MM calculations limited our analysis to three geometries per binding mode, the fact that barrier differences are greater than standard errors within modes provides a layer of confidence to this analysis. Importantly, this stereochemical preferences appears to emerge from the initial substrate conformation rather than FMN positioning, contrasting with mechanism proposed for other photoenzymes.^7,32^

#### Nonadiabatic Effects Drives Chemoselectivity

Our calculations suggest that chemoselectivity between the C-alkylation and XEC may be influenced by nonadiabatic transitions at the geometry **III**, which lies near the conical intersection (Figures 3a and 4). Analysis of the nonadiabatic coupling vector at geometry **III** reveals which atomic displacements could facilitate coupling between the two electronic states (Figure 4b). The figure shows the coupling vector components for each heavy atom in the system, with vector transparency indicating coupling magnitude – opaque vectors represent strong coupling while transparent vectors indicate weak coupling. Most atoms throughout the system show negligible (transparent) components, suggesting that their nuclear motions do not significantly contribute to state crossing. However, important coupling components are visible on atoms involved in C–C bond formation (identified by vector directions with significant projection along the bonding coordinate indicated by a dashed line in Figure 4b), as well as in the atoms associated with α-chloroamide C=O and nitro N=O vibrations.

The correlation between the nonadiabatic coupling and the C–C bond-forming motion suggests that this coordinate participates in the interstate mixing at the conical intersection, potentially facilitating population transfer between electronic states.^33^ Note that the efficiency of nonadiabatic transitions depends on the complete topology of the conical intersection, not solely on the coupling vector alignment.^33^ Additionally, the large energy release upon C-alkylation (ΔE = −1.8 eV between **II** and **IV**) provides a substantial driving force toward this product. The combination of favorable energetics and the participation of C–C coordinate in interstate coupling may together explain why the system preferentially forms alkylation products over XEC intermediates.

XEC requires radical rearrangement from σ* toward the nitro group, forming a nitro-radical anion intermediate necessary for subsequent nitrite dissociation before hydrogen-atom transfer completion^7^ (Figure 3d). This represent a change in excited-state character from the initial α-chloroamide radical state (Figure 3b). We propose that the catalytic preference for C-alkylation may result from the coordinating triad (Y28, R308, R336’) stabilizing **1** binding and potentially inhibiting nitrite dissociation. This hypothesis is supported by two complementary observations: (1) our calculations show that R336’ forms part of the coordination network that hydrogen bond the nitro group, and (2) photoenzymes CsER and GluER-T36A that favor XEC (which requires nitrite dissociation) lack the R336’ residue. We will defer to future work for further computational and experimental validation of this hypothesis.

#### Balanced Electronic and Steric Effects Governs Substrate Selectivity

Having established that photochemical preactivation requires C_α_–Cl bond elongation for proper electron transfer, we investigated whether electronic properties controlling this process determine substrate selectivity in *Gk*OYE-G7. Our electronic-structure calculations on experimentally tested α-halo carbonyl substrates^9^ (Figure 5) reveal that two parameters, electrophilicity and molecular volume, together predict substrate reactivity with high accuracy.

**Figure 5:**
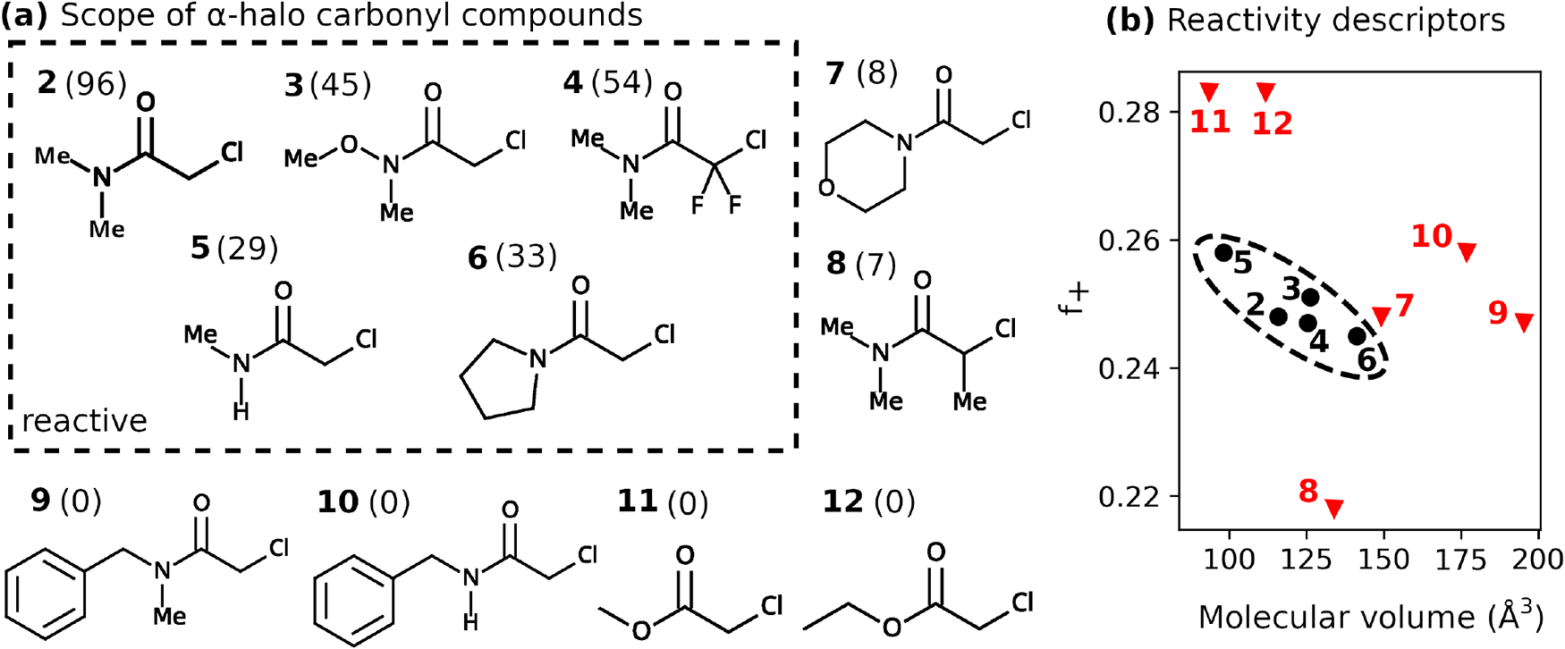
Electronic and steric properties determine substrate selectivity in *Gk*OYE-G7. (a) Substrate scope of α-halo carbonyl compounds with experimental yields (%) shown in parentheses. Dashed line separates reactive (yield >10%) from unreactive substrates. (b) Correlation between electrophilicity (Fukui index *f*_+_) and molecular volume distinguishes reactive (black circles) from unreactive (red triangles) substrates. Dashed oval highlights the optimal reactivity window (*f*_+_ = 0.24–0.26, molecular volume <140 Å^3^).

We employed the Fukui index *f*_+_ ^34^ to quantify electrophilicity at the C_α_ and carbonyl carbons, which constitute the radical center in the CT state and thus control electron transfer efficiency (Figure 3b). Additional calculated properties included C_α_–Cl bond dissociation energy, and molecular volume. We classify substrates exhibiting >10% experimental yield as reactive (values of the complete set of descriptors are in Supporting Information Table S12).

Our analysis reveals that electrophilicity and molecular volume provide useful quantitative guidelines for predicting substrate reactivity (Figure 5b). The empirical thresholds (*f*_+_ = 0.24–0.26, molecular volume <140 Å^3^) define boundaries that distinguish reactive from unreactive substrates. These values serve as practical predictive guidelines, and incorporation of additional factors such as molecular shape and conformational flexibility could further enhance substrate selectivity prediction beyond the trends identified here.

The electrophilicity requirement quantitatively accounts for reactivity differences among structurally similar amides. Tertiary amides **2** and **4** (both *f*_+_ ≈ 0.25) exhibit the highest yields, while **8** (*f*_+_ = 0.22) shows minimal reactivity (7% yield) despite comparable molecular volume. This difference reflects the electron-donating effect of the methyl substituent in **8**, which reduces C_α_ electrophilicity and hinders CT state formation. Conversely, the electron-withdrawing fluorine substituents in **4** optimize electrophilicity for productive photochemistry.

We find that steric constraints explain reactivity losses in bulkier substrates. Amides **9** and **10** (molecular volumes >160 Å^3^) remain unreactive despite optimal electrophilicity, while **6** and **7** (135 and 142 Å^3^, respectively) show lower yields, being at the edge of acceptable volume threshold. The poor reactivity of **7**(8% yield, 142 Å^3^) relative to smaller substrates demonstrates that optimal photoenzyme catalysis requires conformational flexibility beyond simple steric accommodation, as restricted dynamics near the 140 Å^3^ molecular volume threshold limit sampling of productive conformations.

Ester substrates **11** and **12** demonstrate that excessive electrophilicity prevents the reaction. Despite *f*_+_ values >0.28, both esters show zero yields, indicating an optimal rather than minimal electrophilicity requirement. Higher electrophilicity correlates with increased C_α_–Cl bond strength (14 kcal/mol higher dissociation energy for **11** vs **2**, Supporting Information S2.8), creating a kinetic barrier to the halide dissociation step essential for productive photochemistry.

These findings establish design principles for photoenzyme substrate scope engineering. While photoenzyme engineering has utilized mutagenesis to optimize active site steric environment and substrate positioning,^35^ our results suggest that electronic property optimization may represent an underexplored alternative strategy. The identification of specific electrophilicity requirements for productive charge-transfer formation indicates that future engineering efforts, in addition to introducing mutations to tune the sterics of the active site, could potentially target the electrostatics of the active site to modulate the electronic properties of the substrate(s) and the cofactor to achieve desired photochemical reactivity.

#### Complete Mechanistic Picture of Triple Selectivity

The above integrated findings demonstrate that *Gk*OYE-G7 achieves simultaneous control of all three selectivity types through distinct molecular mechanisms: preactivation requirements likely determine substrate scope, binding mode barriers control enantioselectivity, and strong nonadiabatic coupling effects, combined with a large driving force, favor chemoselectivity toward C-alkylation (Figure 6). This multilevel control represents a sophisticated solution to the challenge of selective radical chemistry, with the preactivation mechanism providing a framework for understanding broader substrate selectivity patterns.

**Figure 6:**
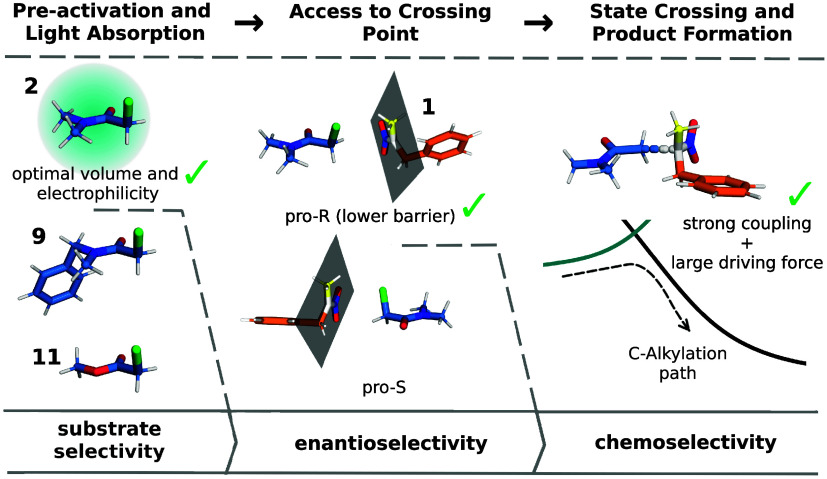
Overview of the triple selectivity control in *Gk*OYE-G7: Substrate selectivity is controlled by the preactivation step involving C_α_-Cl bond elongation that enables productive light absorption, requiring appropriate molecular volume and electrophilicity (exemplified by substrate **2**). Enantioselectivity likely arises from the different energy requirements to reach the crossing point in pro-R and pro-S binding modes. Chemoselectivity is controlled by strong nonadiabatic coupling and substantial driving force at the crossing point that favor C-alkylation over alternative pathways.

## Conclusion

We have established that photoenzyme selectivity operates through reaction-level control rather than traditional binding-level selectivity, revealing fundamental principles that govern radical chemistry in photoenzymatic systems. Using *Gk*OYE-G7 as a model system, we demonstrate that photoenzyme active sites accommodate substrates in multiple conformations with similar energies, preventing traditional binding-based selectivity models from fully explaining stereochemical control. Importantly, productive photochemistry requires a preactivation mechanism involving C_α_–Cl bond elongation that creates electronic compatibility for charge-transfer state formation, providing the mechanistic basis for understanding photoenzyme substrate scope. Substrate selectivity emerges from electronic-steric criteria that balance optimal electrophilicity with appropriate molecular volumes, establishing predictive guidelines to rationalize substrate scope. Meanwhile, enantioselectivity likely arises from differential reaction barriers among substrate binding modes rather than preferential binding, and chemoselectivity is likely driven by a conical intersection that directs the reaction pathway through favorable coupling and thermodynamic driving forces. These computational insights complement existing binding-based selectivity models^17^ and provide design principles for engineered photoenzymes with predictable selectivity profiles, expanding the toolkit for rational biocatalyst development.

## Computational Methods

### System Setup and Parametrization

An all-atom model of *Gk*OYE-G7 was constructed from the wild-type crystal structure (PDB: 3GR7)^36^ with mutations D73C, A104H, and Y264W introduced manually using PyMOL.^37^ The system was assembled as a homotetramer of 1360 amino acids consistent with its biological oligomeric state.^25,36^ Protonation states for standard MD simulations were determined using PROPKA 3.5.0 ^38^ at pH 9.0, with FMN cofactor modeled in its fully reduced anionic hydroquinone form, while constant pH MD simulations employed dynamic protonation states (see Constant pH MD section below). Substrates 2-nitropropylbenzene nitronate and 2-chloro-*N,N*-dimethylacetamide were docked into the active site using AutoDock Vina 1.2.0.^39^ The complete system was solvated in a cubic water box (136.9 Å sides) with TIP3P^40^ water molecules and neutralized with sodium ions, resulting in a simulation box with a total of 263,341 atoms. Custom CHARMM36-compatible force field^41^ were derived for both substrates using a genetic algorithm optimization approach to reproduce quantum mechanical reference calculations (SI Section S2.1).

### Molecular Dynamics Simulations

All MD simulations were performed using GROMACS 2022.5 ^42^ with a 2 fs time step, except for constant pH MD simulations which employed a modified GROMACS 2024 version. Bond constraints involving hydrogen atoms were applied using LINCS^43^ for the protein and SHAKE^44^ for the solvent. Long-range electrostatics interactions were treated using Particle-Mesh-Ewald (PME)^45^ with a 1 nm real-space cutoff, while van der Waals interactions used a 1 nm cutoff with dispersion corrections. Periodic boundary conditions were applied in all three dimensions. Temperature was maintained at 300 K using the Nose-Hoover thermostat^46^ and pressure controlled at 1 bar using the Parrinello–Rahman barostat.^47^ Minimization was conducted with Steepest Descent algorithm until *F*_max_ < 1000 kJ mol^–1^ nm^–1^. Equilibration was conducted stepwise in the NPT ensemble. An initial 100 ns simulation was performed with harmonic positional restraints applied on heavy atoms of the protein, cofactor and substrates. The restraint force was gradually reduced from 100 to 0 kJ mol^–1^ nm^–2^ over the equilibration, decreasing by 10 kJ mol^–1^ nm^–2^ every 10 ns to allow smooth structural relaxation of the manually introduced mutations and docked substrates without introducing distortions. Following the restrained equilibration, an additional 100 ns of unconstrained simulation was performed to ensure complete thermal and pressure equilibration of the system. Three independent simulations were performed using different random velocity seeds to provide uncertainty estimates, reported as mean ± standard error.

### Bias-Exchange Metadynamics

Enhanced sampling of substrate binding was achieved through bias-exchange metadynamics^19^ using GROMACS patched with PLUMED 2.9.0.^48^ To explore substrate binding pathways while preventing diffusion into bulk solvent, we employed a funnel-shaped restraint potential that guides substrates from the surrounding solvent toward the enzyme active site (Figure 7).^49^ The funnel geometry consists of a cylindrical region (radius 1 Å) transitioning at 32 Å to a conical section with an opening angle of 0.45 radians, oriented along an axis defined by reference points in the rigid β-barrel domain.

**Figure 7:**
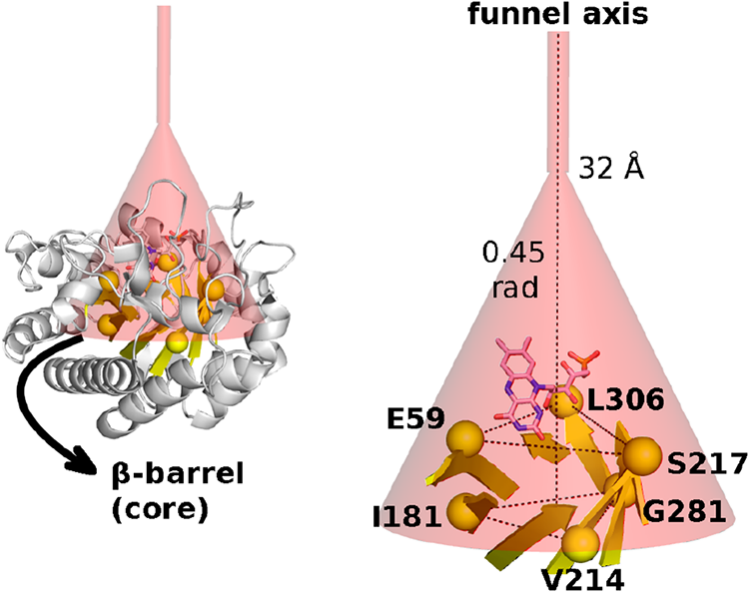
Funnel restraint potential used to define substrate binding collective variable. The funnel axis is defined by C_α_ centroids of residues I181/V214/G281 (protein core, 0 Å) and E59/S217/L306, pointing toward solvent and passing through FMN. These residues are located in the rigid β-barrel domain (shown in yellow) to provide a stable coordinate reference. The geometry transitions from cylindrical radius 1 Å to conical (0.45 rad opening) at 32 Å.

Standard binding free energies were calculated from the resulting free energy profiles (Figures 2a and S10) using an analytical correction for the funnel restraints.^49^ The corrected binding constant was first computed as

1 $$K_{b}=C^{0}\pi R_{\mathrm{cyl}}^{2}\int \mathrm{dz}e^{-\beta \left[\Delta W\left(z\right)\right]}$$

where *C* ^0^ is the standard concentration (1/1661 Å^–3^), *R*_cyl_ is the cylindrical radius (1 Å), *z* is the distance coordinate along the funnel axis, Δ*W*(*z*) is the change in free energy along the funnel axis with 0 set to bulk solvent, and β = 1/*k*_B_ *T*. The standard binding free energy was then obtained as Δ*G*_bind_^°^ = −*k*_B_ *T*ln­(*K*_b_).

Seven collective variables were employed in bias-exchange: two tracking each substrate’s position along this funnel axis (0 Å = protein core, > 35 Å = solvent, as shown in Figure 2a), one monitoring flexible loop motion near the active site, and four measuring distances between the nitronate group and key binding residues. Eight replicas were simultaneously evolved for 500 ns each (4 μs cumulative sampling per run), with seven using biasing potentials on different collective variables and one maintaining unbiased sampling as reference. Well-tempered metadynamics^50^ with Gaussian hills deposited every 5 ps (initial height 0.5 kJ/mol) was employed, with replica exchanges attempted every 5 ps following the Metropolis criterion^51^ (detailed definitions in Supporting Information Section S1.1). Convergence was assessed through analysis of binding free energy profiles and collective variable trajectories (SI Section S2.2).

### Constant pH Molecular Dynamics

CpHMD were performed with a modified GROMACS 2024 implementation^52^ with the CHARMM36m-lambdadyn force field. The key modifications from standard MD include: (1) Y28 was treated as titratable residue with dynamic switching between protonated and deprotonated states, (2) a buffer site (modified water molecule) was placed 3.0 nm from Y28 to maintain charge neutrality during protonation changes, and (3) the Fast Multipole Method (FMM) electrostatics solver was employed instead of PME.^52^ Three catalytically competent geometries were simulated for 100 ns each at pH 9.0, with protonation states recorded every 20 ps.

### Multireference QM/MM Calculations

QM/MM calculations were performed using ORCA 6.0.1 ^53^ with electrostatic embedding to investigate the photochemical reaction mechanism. The QM region (68 atoms, charge −2) comprised both substrates and the FMN isoalloxazine ring with adjacent CH_2_ group from the ribitol-phosphate moiety. A hydrogen link atom was positioned at the QM-MM boundary with charge-shift scheme to prevent overpolarization. State-averaged complete active space self-consistent field (CASSCF)­(4,4)/def2-TZVPP^31,54,55^ calculations over 4 roots were employed to capture the multiconfigurational diradical character arising from C–Cl bond cleavage. Dynamic correlation was incorporated through second-order Dynamic Correlation Dressed CAS (DCD-CAS(2))^56^ corrections. CASSCF calculations utilized resolution of identity^57^ and chain-of-sphere exchange approximations^58^ for computational efficiency. Active space selection rationale is provided in SI Sections S1.2 and S1.3. Nonadiabatic coupling vectors were calculated using PySCF 2.10.0 ^59^ at the SA-CASSCF level.

### Reaction Path Calculations

Initial geometries from the classical MD simulations of catalytically competent binding poses were first optimized at MM level. For subsequent reaction path determination at the QM/MM level, full multiconfigurational geometry optimization along reaction coordinates was computationally prohibitive, and conventional single-reference SCF procedures often failed to converge at diradical geometries where the HOMO–LUMO gap becomes very small. Therefore, we employed ground state finite-temperature DFT (PBEh-3c functional^60^), with Fermi’s smearing (default parameters), which stabilizes SCF convergence in these challenging regions through fractional orbital occupations compared to standard DFT.^61^ This approach can capture the electronic state evolution from closed-shell reactant through diradical intermediate to closed-shell product along the reaction coordinate (SI Section 2.7).

To determine reaction paths, first, potential energy surface scans along the alkylation C–C bond lengths identified appropriate product geometries, followed by full optimization of reactant and product end points for the QM region only. Nudged elastic band (NEB) calculations^62^ then connected these end points to generate optimized reaction paths. Finally, the local MM environment (atoms within 15 Å of the QM region) was relaxed while constraining the QM atom positions to their NEB-determined coordinates.

### Electronic Descriptor Calculations

Substrate molecular descriptors were calculated using ORCA 6.0.1 ^53^ in implicit CPCM water solvent.^63^ Electrophilicity Fukui indexes (*f*_+_)^34^ were computed at ωB97X/def2-TZVPP^64^ level using Hirshfeld charges,^65^ while bond dissociation energies employed coupled-cluster DLPNO–CCSD­(T)/def2-TZVPP^66^ with TightPNO thresholds and counterpoise correction for basis set superposition error. With our largest substrate **9** containing only 25 atoms and using the most conservative PNO criteria available, DLPNO–CCSD­(T) should provide reasonable bond dissociation energies with minimal approximation errors.^67^

## Supplementary Material



## Notes

Supporting methods including definition of collective variables; CASSCF active space selection and MO overlap analysis; force field parametrization data for substrates; convergence analysis of bias-exchange metadynamics simulations; protein flexibility analysis; binding free energy calculations; CpHMD results; QM/MM validation studies; individual reaction pathway calculations for different binding modes; and substrate molecular descriptors (PDF)
