# Supplementary material for: Molecular Origins of Simultaneous Chemo‑, Enantio‑, and Substrate Selectivity in Non-Natural Photoenzymatic Radical Reactions
Source: J Am Chem Soc. 2025 Oct 30;147(45):41639–49. doi: 10.1021/jacs.5c12802 (PMC12616699; doi:10.1021/jacs.5c12802)
Supplement: Supplementary file 1 [file ja5c12802_si_001.pdf]

Supporting Information

Molecular Origins of Simultaneous Chemo-, Enantio-, and Substrate Selectivity in Non-Natural Photoenzymatic Radical Reactions

Felipe Curtolo,[†] Sijia S. Dong*,[†,‡]

Contents

S1. Supporting Methods **S2**

S1.1. Collective variables definition . . . S2

S1.2. CASSCF active-space selection . . . S2

S1.3. Overlap analysis of active orbitals . . . S4

S2. Supporting Results **S5**

S2.1. Substrates force-field parametrization . . . S5

S2.2. Bias-exchange metadynamics convergence . . . S9

S2.3. Loop flexibility analysis . . . S11

S2.4. Filtering MD trajectories to obtain catalytic poses . . . S11

S2.5. Binding free energy of the α-chloroamide **2** . . . S12

S2.6. Probing Y28 protonation state with constant pH MD simulations . . . S12

S2.7. QM/MM validation . . . S13

S2.8. Individual reaction paths . . . S15

S2.9. Substrate steric and electronic descriptors and volume calculations . . . S15

S3. Supporting References **S16**

† Department of Chemistry and Chemical Biology, Northeastern University, Boston, Massachusetts 02115, USA

‡ Department of Physics and Department of Chemical Engineering, Northeastern University, Boston, Massachusetts 02115, USA

* Corresponding author: Sijia S. Dong, s.dong@northeastern.edu

S1. Supporting Methods

S1.1. Collective variables definition

A total of 8 replicas were employed in the bias-exchange metadynamics simulations, with each replica biasing a different collective variable (CV) related to substrate binding. The definition of each CV with their respective Gaussian width (σ), bias factor and boundaries are specified in Table S1.

Table S1. Definition and parameters of collective variables used in bias-exchange metadynamics.

Index	Definition	σ (nm)	Bias factor (K)	CV boundaries (nm)
CV1	projection of nitroalkane **1** along funnel axis[a]	0.05	50	0.0 – 6.0
CV2	projection of α-chloroamide **2** along funnel axis[a]	0.05	50	0.0 – 6.0
CV3	distance loop-6[b] to FMN-N10	0.05	30	0.0 – 4.0
CV4	distance of nitroalkane **1**-N to K109-N_ζ	0.04	55	0.0 – 6.2
CV5	distance of nitroalkane **1**-N to R257-C_ζ	0.04	55	0.0 – 6.2
CV6	distance of nitroalkane **1**-N to R308-C_ζ	0.04	50	0.0 – 6.2
CV7	distance of nitroalkane **1**-N to R336'-C_ζ	0.04	50	0.0 – 6.2

[a] Funnel potential used to constraint substrate position.
[b] Most flexible loop connecting α-helix 6 to β-strand 6.

Two types of collective variables were employed: CV1 and CV2 are projection CVs that measure substrate position along an axis used to define a funnel potential[1] that restraints the substrates within the active site. CV3-CV7 are distance-based collective variables.

The funnel potential was defined using two reference points located in the rigid β-barrel domain: point 1 as the centroid of the C_α atoms of residues I101, V214, and G281, and point 2 as centroid of the C_α atoms of residues E59, S217, and L306 used to define the funnel axis, as shown in the main text Fig. 7. The funnel geometry consisted of a cylindrical region (radius 1 Å) that transitions to a conical section at funnel axis = 32 Å with an opening angle of 0.45 radians. The bias was applied over a range of 5 to 52 Å along the funnel axis with a force constant of 35,100 kJ/mol·nm^2, effectively restraining the substrates within the funnel while allowing exploration of binding pathways.

CV3 involves loop-6 (S249–Q265), the longest and most flexible loop of *Gk*OYE-G7, which we identified as crucial for substrate binding. This loop acts as a hinge that can close or expose the active site during the binding process. CV3 was defined as the distance between the center of mass of C_γ and C_δ atoms of residues I253 and V254 (belonging to loop-6) and the N10 atom of the FMN cofactor.

CV4–CV7 represent direct pairwise distances between the nitrogen atom of the nitronate group in substrate **1** and the nearest positively charged residues: K109 (CV4), R257 (CV5), R308 (CV6), and R336' from the adjacent protein chain (CV7). These interactions are expected to be important for substrate binding and orientation within the active site.

S1.2. CASSCF active-space selection

CASSCF calculations were necessary to study the C-alkylation reaction between **1** and **2** because single-reference methods fail to provide even qualitatively correct zeroth-order wavefunctions in some geometries (discussed in Section S2.7). When the C_α–Cl bond breaks, the lowest energy electronic state is a singlet open-shell diradical with one unpaired electron on FMN (neutral semiquinone) and another at **2**-C_α (see main text, Fig. 3b) This electronic structure is inherently multiconfigurational due to two possible spin-coupling arrangements of the unpaired electrons (↑ / ↓ or ↓ / ↑ in Fig. S1b).

The CASSCF active space was constructed starting from geometry **II** (Main text, Fig. 3c), which was expected to exhibit the highest multiconfigurational diradical character. Initial orbitals were obtained from broken-symmetry unrestricted QM(DFT)/MM calculations using ωB97X/def2-SV(P)[2,3]. While this approach yields

spin-contaminated wavefunctions, it provides appropriate diradical starting orbitals that would be inaccessible using restricted closed-shell calculations.

The two singly occupied molecular orbitals (MOs) displayed expected shapes: one resembling a π orbital on FMN (π_{FMN}) and the other a non-bonding orbital on substrate **2** (n), forming an initial CAS(2,2) (Fig. S1c). To include MOs involved in bond formation during the nucleophilic attack of the nitronate group at the electron-deficient radical C_α (Fig. S1a), we identified the doubly occupied π orbital of the $N = C$ nitronate bond and its corresponding antibonding partner ($\pi_{\mathrm{C=N}}$ and π^*) through visual inspection and orbital rotation, expanding to our final active space CAS(4,4) (Fig. S1c).

To assess the importance of extended conjugation, we tested a larger CAS(8,6) active space that included additional nitro group valence orbitals (π_{nitro} and n_{nitro} in Fig. S1c). However, these orbitals showed occupation numbers of ~ 2.00, indicating minimal contribution to the multiconfigurational character. Additional FMN orbitals were excluded based on previous flavin studies showing their involvement primarily in high-energy excitations[4].

The appropriateness of the chosen CAS(4,4) active space was verified throughout the entire reaction coordinate. As shown in Figure 3c of the main text, the active orbitals smoothly change their chemical character and exhibit consistent multiconfigurational behavior along the reaction path, with occupation numbers demonstrating significant deviation from purely closed-shell (occ=2.00 or 0.00) at critical points of the reaction.

Basis set projection from def2-SV(P) to def2-TZVPP was performed after CASSCF convergence in the smaller basis set. For adjacent geometries along the reaction path, previously converged CASSCF orbitals were used sequentially as initial guesses.

(a) C-C bond formation

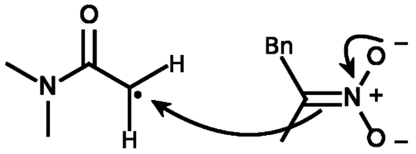

(b) CASSCF(8,6): CI expansion

Determinant	Weight (%)
2-2-2-↑-↓-0	46.95
2-2-2-↓-↑-0	46.95
2-2-0-↑-↓-2	2.56
2-2-0-↓-↑-2	2.56

(c) Tested active spaces

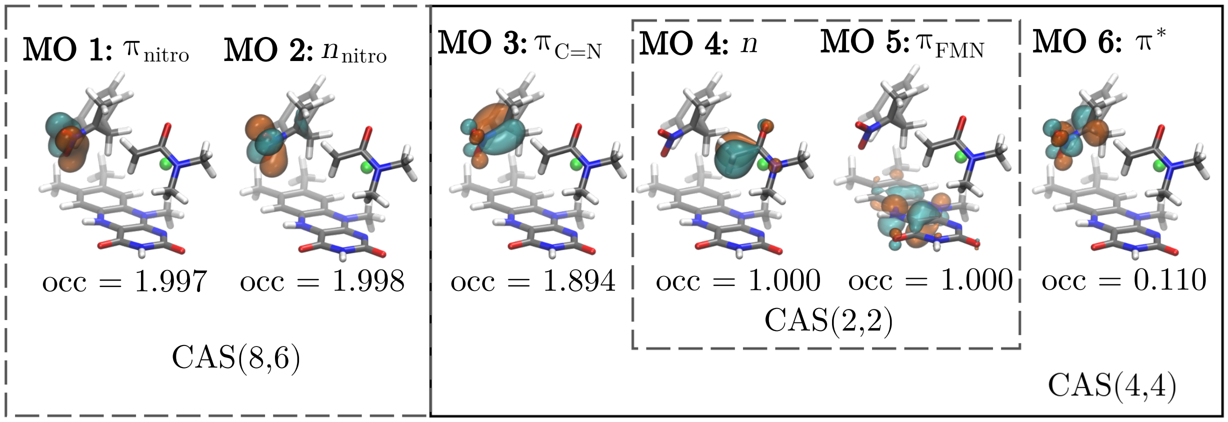

Figure S1. Active space selection at diradical geometry **II**. (a) Chemical intuition from C–C bond formation mechanism guided active space selection. (b) Configuration Interaction (CI) expansion for ground-state/diradical showing multiconfigurational character with dominant diradical configurations (↑ / ↓ and ↓ / ↑ pairs). (c) Occupation numbers for different active spaces. MOs 1–2 have occupation ~ 2.0, justifying exclusion from the final CAS(4,4).

S1.3. Overlap analysis of active orbitals

A critical concern in CASSCF calculations along reaction coordinates is maintaining orbital consistency to ensure meaningful energy profiles and avoid artificial discontinuities. Orbital rotations between geometries can lead to sudden changes in active space character, potentially causing unphysical energy jumps or incorrect description of the reaction mechanism. To validate our CASSCF(4,4) calculations for the C-alkylation reaction between nitronate **1** and α-chloroamide **2**, we analyzed the spatial evolution and overlap of the four active orbitals throughout the reaction path.

The overlap plots confirm active space stability throughout the reaction coordinate (Fig. S2). All four active orbitals maintain overlap values above 0.5 between adjacent geometries, with no sudden drops that would indicate problematic orbital rotations or discontinuities. MOs 5 and 6 exhibit consistently high overlaps (≥ 0.9), demonstrating these orbitals retain their character throughout the reaction.

For MOs 3 and 4, which show overlap minima near 0.6 at specific geometries, we employed ridgeline plots to show what was happening at these geometries. MO 3 undergoes a smooth, chemically expected transition from nonbonding to bonding character as the C–C bond forms. Similarly, MO 4 shows a gradual evolution from antibonding to nonbonding and back to antibonding character. Importantly, the ridgeline plots demonstrate that even when overlaps drop to 0.6, the orbital transformations are continuous and physically meaningful rather than abrupt rotations. These results validate that our CASSCF(4,4) active space remained stable and physically meaningful throughout the entire reaction path.

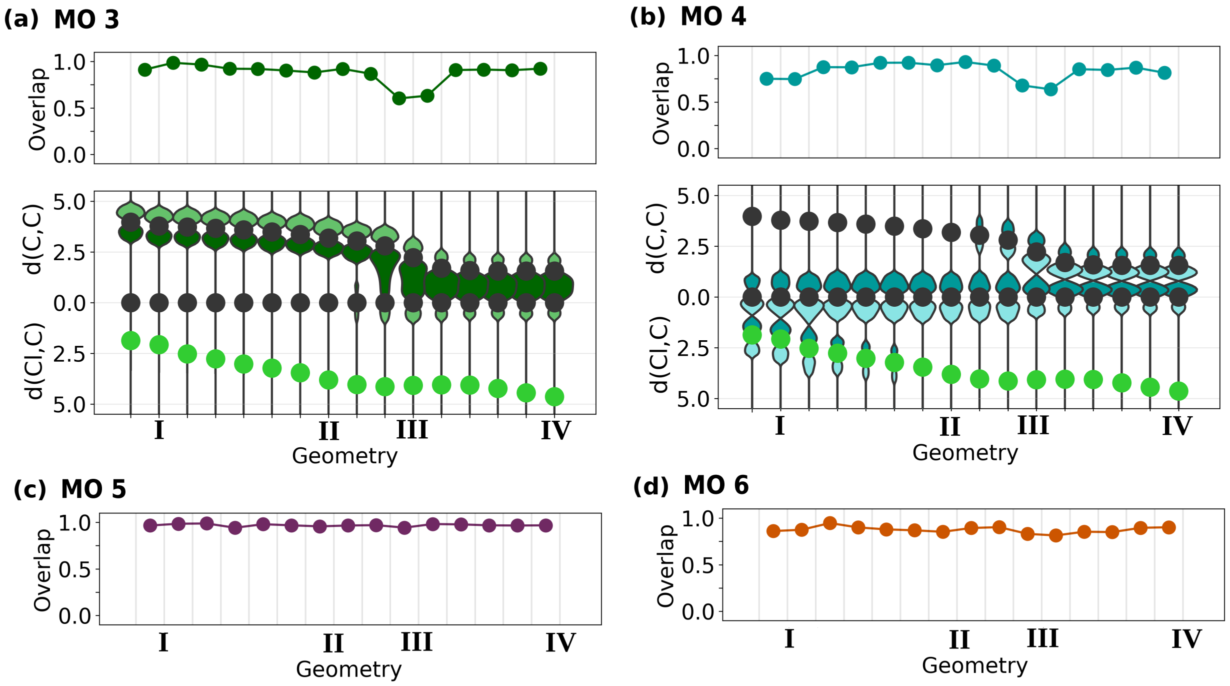

Figure S2. Overlap analysis demonstrating CASSCF(4,4) active space stability along the C-alkylation reaction pathway. Each panel (a-d) shows molecular orbitals 3-6 (see Fig. S1 for MO indexes), respectively, with overlap values between adjacent geometries confirming orbital continuity throughout the reaction. Panels (a) and (b) include additional ridgeline plots in the lower subpanels for MOs 3 and 4, where each ridge represents the MO projected onto the bond-forming axis. Roman numerals I-IV correspond the structures discussed in the main text.

Orbital overlaps between adjacent geometries were calculated after aligning the geometries to minimize translational and rotational effects. For the ridgeline visualization, cube files containing the 3D orbital amplitudes φ(x,y,z) were generated for each geometry. These were then projected onto the bond-forming axis by integrating φ(x,y,z) to yield a 1D function φ(d) where d represents the position along the bond axis.

S2. Supporting Results

S2.1. Substrates force-field parametrization

Proper treatment of intermolecular interactions and intramolecular torsional degrees of freedom in the MD simulations requires an accurate force field. While the CHARMM General Force Field (CGenFF) [5] provides parameters for many organic functional groups and allows direct derivation of ligand CHARMM-potential parameters (Eq. S1), it performs poorly for the α-chloroamide **2** and lacks specific parameters for the nitronate **1**, instead borrowing parameters from nitroalkanes.

$$\begin{aligned} V = &\sum_{\text{bonds}} k_b(b-b_0)^2 + \sum_{\text{angles}} k_\theta(\theta-\theta_0)^2 + \sum_{\text{Urey-Bradley}} k_{\text{UB}}(r_{1,3}-r_{1,3;0})^2 + \\ &\sum_{\text{dihedrals}} k_\phi[1+\cos(n\phi-\phi_0)] + \sum_{\text{impropers}} k_\varphi(\varphi-\varphi_0)^2 + \\ &\sum_{\text{nonbondeded}} \left(\frac{q_i q_j}{\varepsilon r_{ij}} + \epsilon_{ij}\left[\left(\frac{R_{\text{min},ij}}{r_{ij}}\right)^{12} - 2\left(\frac{R_{\text{min},ij}}{r_{ij}}\right)^{6}\right]\right) \end{aligned} \tag{S1}$$

To address these limitations, ligand-specific parametrization was performed to reproduce the accuracy of reference quantum mechanical (QM) calculations. Following standard CHARMM protocols [5], substrate geometries were optimized at the MP2/6-31G(d) level for **2** and at MP2/6-31+G(d) for **1** (due to its anionic character). Interaction curves were obtained using TIP3P water molecules [6,7] with HF/6-31G(d) energies, while torsion energies were calculated at the MP2/aug-cc-pVTZ level.

Parameter optimization was conducted using a genetic algorithm [8] implemented in a local Python script using NumPy v1.19.5 for random number generation, with mean squared error as the fitness function. The algorithm employed a population size of 100 individuals over 200 generations, with each candidate using 24 bits per parameter. Random numbers were used to generate the initial population and to control selection, crossover, and mutation. Parent selection used tournament selection with tournament size k=3. Offspring generation employed one-point crossover with probability 0.9 and bit-flip mutation with per-bit probability $1/(24 \times \text{number of parameters})$. Multiple optimization cycles were performed with varying parameter ranges: initially using large-spaced intervals, then progressively increasing precision with finer grid intervals as improved parameters were obtained. The number of optimized parameters differed for each substrate reflecting their distinct chemical characteristics and force field requirements (see Tables S2-S10).

Figures S3 and S4 show interaction curves with water and torsion energies for **2** and **1**, respectively. The optimized parameters enable the force field to reproduce reference QM calculations with high accuracy. The main discrepancies occur in high-energy regions (such as short interaction distances or near transition states), while all minimum energies and distances are well-reproduced. Therefore, the optimized parameters represent a significant improvement over default CGenFF and enable construction of more accurate force fields for the substrates.

To validate the derived force fields beyond the fitted interaction and torsion energies, independent geometry optimizations were performed. For α-chloroamide **2**, an RMSD of 0.074 Å was observed, while for nitronate **1**, the RMSD was 0.033 Å. These results confirm that the optimized parameters accurately reproduce both energetics and geometries.

The optimized parameters for **2** are reported in Tables S2–S4, while **1** parameters are provided in Tables S5–S12. All remaining parameters not explicitly reported here remain unchanged from the original CGenFF [5].

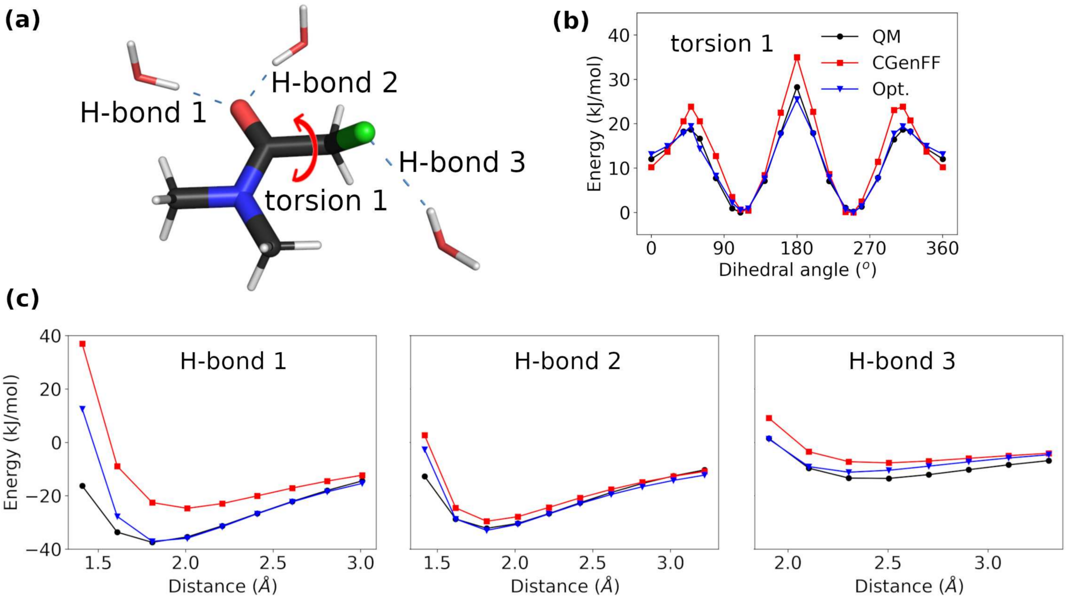

Figure S3. CHARMM force field parametrization of α-chloroamide **2** (a). Torsion energies (b) and interaction energies with water (c) are shown in three levels of theory: reference quantum mechanical energy QM in black; original CGenFF force field in red; and after optimization in blue.

Table S2. Optimized CHARMM-compatible charges for α-chloroamide **2**.

Atom Index*	Atom Type	Charge (a.u)
1	CLGA1	-0.157
2	CG321	0.577
3	HGA2	0.090
4	HGA2	0.090
5	CG2O1	0.351
6	OG2D1	-0.587
7	NG2S0	-0.730
8	CG331	-0.087
9	HGA3	0.090
10	HGA3	0.090
11	HGA3	0.090
12	CG331	-0.087
13	HGA3	0.090
14	HGA3	0.090
15	HGA3	0.090

*Displayed in Fig. S5(a)

Table S3. Optimized CHARMM-compatible angle parameters for α-chloroamide **2**.

Atom Types	k_θ (kJ.mol^{-1}rad^{-2})	θ_0 (°)
CG2O1 – CG321 – CLGA1	271.960	111.82

Table S4. Optimized CHARMM-compatible proper dihedral parameters for α-chloroamide **2**.

Atom Types	k_ϕ (kJ.mol^{-1})	ϕ_0 (°)	n
NG2S0 – CG2O1 – CG321 – CLGA1	1.405805	180.0	3
OG2D1 – CG2O1 – CG321 – CLGA1	0.272320	180.0	1
OG2D1 – CG2O1 – CG321 – CLGA1	2.930346	180.0	2
OG2D1 – CG2O1 – CG321 – CLGA1	1.219815	180.0	3

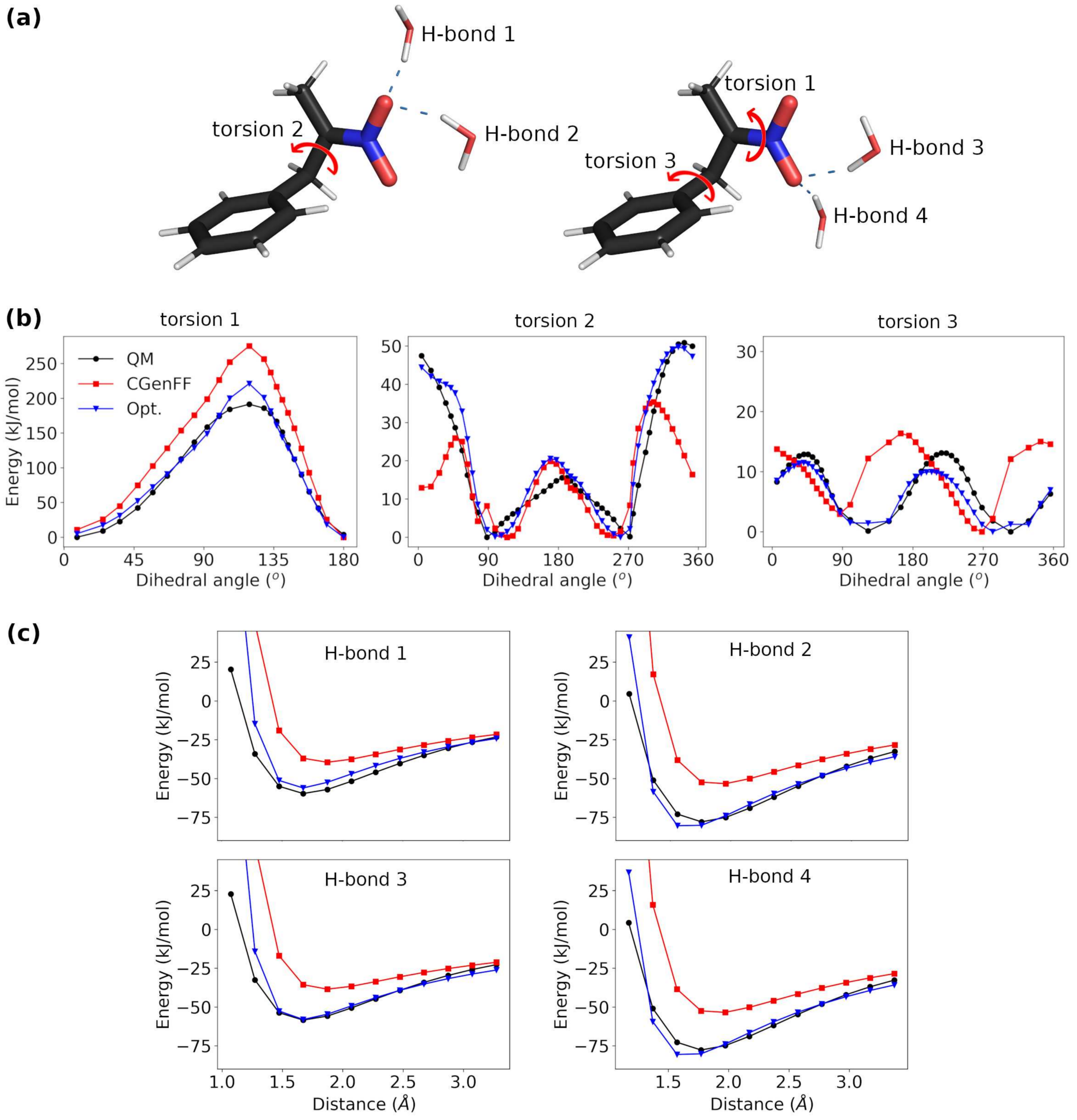

Figure S4. CHARMM force field parametrization of nitronate **1** (a). Torsion energies (b) and interaction energies with water (c) are shown in three levels of theory: reference quantum mechanical energy QM in black; original CGenFF force field in red; and after optimization in blue.

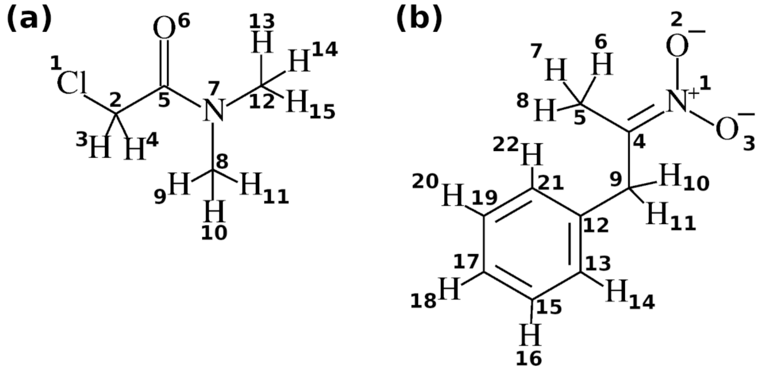

Figure S5. Atoms indexes of α-chloroamide **2** (a) and nitronate **1** (b) to reference the charge parameters reported in Tables S2 and S5.

Table S5. Optimized CHARMM-compatible charges for nitronate **1**.

Atom Index*	Atom Type	Charge (a.u)
1	NG2O2	0.225
2	OG2N2	-0.451
3	OG2N2	-0.451
4	CG2D1	-0.970
5	CG331	0.155
6	HGA3	0.090
7	HGA3	0.090
8	HGA3	0.090
9	CG321	0.042
10	HGA2	0.090
11	HGA2	0.090
12	CG2R61	0.000
13	CG2R61	-0.115
14	HGR61	0.115
15	CG2R61	-0.115
16	HGR61	0.115
17	CG2R61	-0.115
18	HGR61	0.115
19	CG2R61	-0.115
20	HGR61	0.115
21	CG2R61	-0.115
22	HGR61	0.115

*Displayed in Fig. S5(b)

Table S6. Optimized CHARMM-compatible Lennard-Jones parameters for nitronate **1**.

Atom Type	ϵ (kJ.mol^{-1})	R_{min} (nm)
NG2O2	0.0488924	0.278010644
OG2N2	0.5797042	0.279851967

Table S7. Optimized CHARMM-compatible bond parameters for nitronate **1**.

Atom Types	k_b (kJ.mol^{-1}nm^{-2})	b_0 (nm)
CG2D1 – NG2O2	196648.00	0.1283
NG2O2 – OG2N2	242672.00	0.1225

Table S8. Optimized CHARMM-compatible angle parameters for nitronate **1**.

Atom Types	k_θ (kJ.mol^{-1}rad^{-2})	θ_0 (°)
CG321 – CG2D1 – NG2O2	196.648	125.6
CG331 – CG2D1 – NG2O2	196.648	125.6
CG2D1 – CG321 – CG2R61	125.520	114.0
CG2D1 – NG2O2 – OG2N2	280.328	123.6
OG2N2 – NG2O2 – OG2N2	439.320	128.0

Table S9. Optimized CHARMM-compatible proper dihedral parameters for nitronate **1**.

Atom Types	k_ϕ (kJ.mol^{-1})	ϕ_0 (°)	n
CG331 – CG2D1 – CG321 – CG2R61	0.000000	180.0	3
NG2O2 – CG2D1 – CG321 – HGA2	0.024035	180.0	3
NG2O2 – CG2D1 – CG321 – CG2R61	1.679244	180.0	1
NG2O2 – CG2D1 – CG321 – CG2R61	7.572831	180.0	2
NG2O2 – CG2D1 – CG321 – CG2R61	0.063451	180.0	3
NG2O2 – CG2D1 – CG321 – CG2R61	0.098454	180.0	4
NG2O2 – CG2D1 – CG331 – HGA3	0.627600	180.0	3
CG321 – CG2D1 – NG2O2 – OG2N2	19.242401	180.0	2
CG331 – CG2D1 – NG2O2 – OG2N2	19.242401	180.0	2
CG2R61 – CG2R61 – CG321 – CG2D1	1.289440	157.0	2

Table S10. Optimized CHARMM-compatible improper dihedral parameters for nitronate **1**.

Atom Types	k_φ (kJ.mol^{-1}rad^{-2})	φ_0 (°)
NG2O2 – OG2N2 – OG2N2 – CG2D1	224.2624	0.0

S2.2. Bias-exchange metadynamics convergence

Sampling convergence was evaluated through analysis of the demultiplexed trajectories of collective variables CV1 and CV2, reflecting binding of substrates 1 and 2 respectively (Section S1.1), which demonstrated extensive sampling of the configurational space with multiple physical transitions observed between bound and unbound states (colored blue and yellow in Figure S7). The demultiplexing procedure was applied to recover continuous physical trajectories from the replica-exchange simulation, ensuring that the observed transitions represent genuine binding/unbinding events rather than artifacts of the exchange process.

In addition, the overall shape of the free energy surface, including the position and depth of binding minima, were consistent across the three independent runs, indicating similar exploration of the free energy landscape.

Free energy convergence was evaluated by comparing binding free energy profiles calculated from different cumulative sampling windows. When analyzing data from 400 ns per replica (3.2 μs total sampling across 8 replicas), $\Delta G^{\circ}_{\text{bind}}$ averaged 3.37 ± 1.02 kcal/mol across the three independent runs. Extension to 500 ns per replica (4.0 μs total sampling time) yielded 2.92 ± 0.35 kcal/mol, representing convergence within 0.45 kcal/mol between sampling windows (Fig. S6). The reduction in uncertainty (from 1.02 to 0.35 kcal/mol) combined with consistent mean values that remained within overlapping uncertainty ranges, demonstrates that the simulations achieved adequate sampling for reliable free energy calculation.

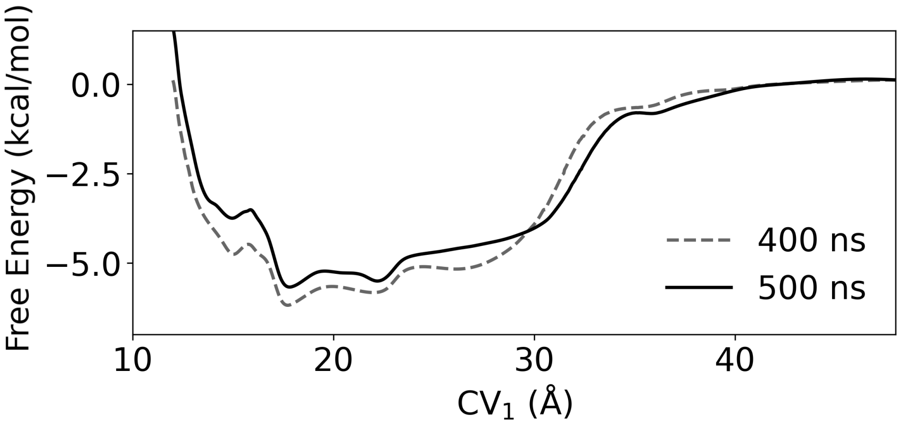

Figure S6. Free energy convergence analysis for binding of substrate 1 in *Gk*OYE-G7. Free energy profiles calculated from 400 ns (dashed line) and 500 ns (solid line) of sampling replica, averaged over $n = 3$ independent bias-exchange metadynamics simulations. The small difference between profiles demonstrates convergence of the binding free energy calculation.

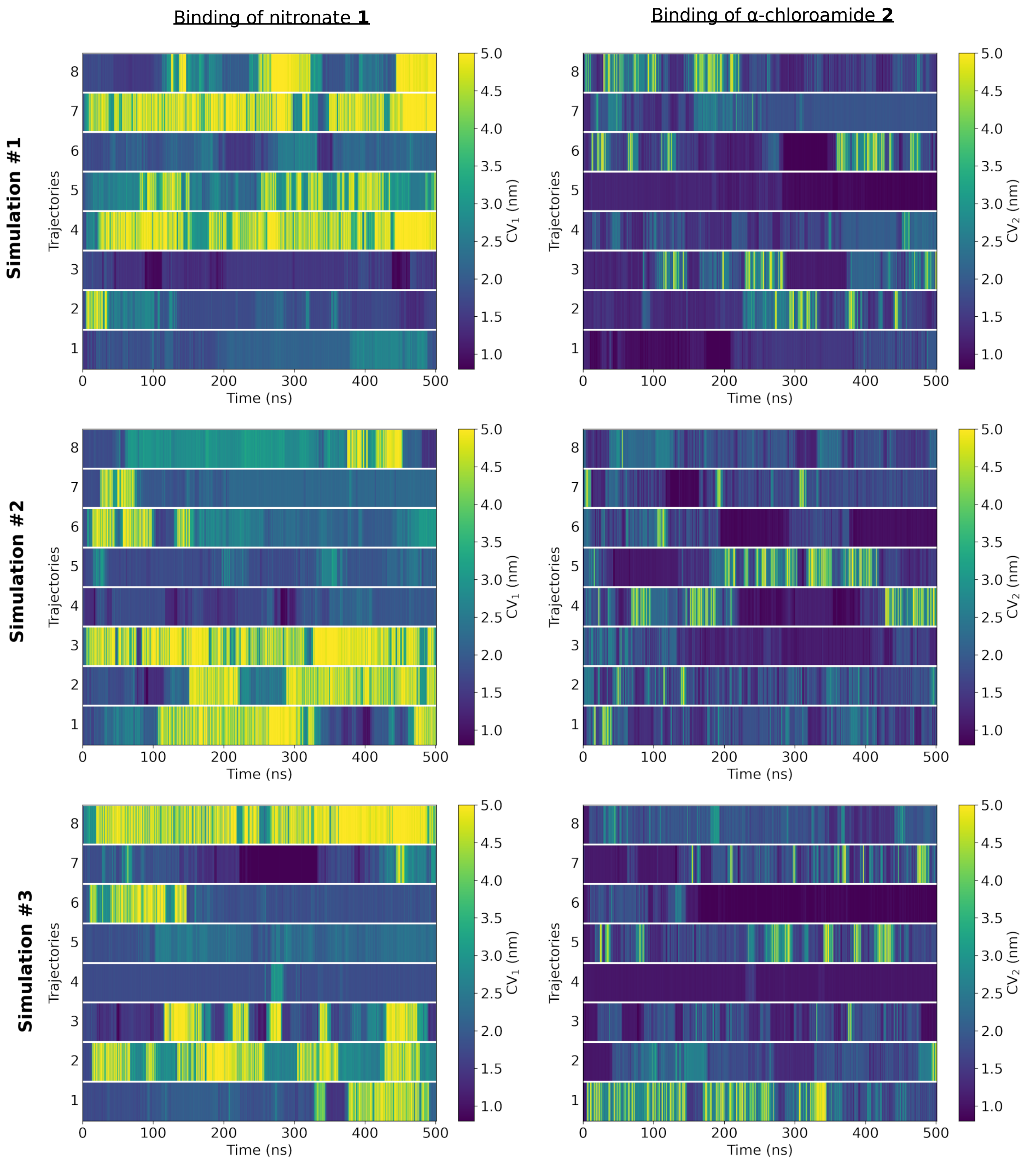

Figure S7. Sampling convergence analysis from demultiplexed collective variable trajectories. Time evolution of collective variables CV1 (nitronate **1** binding, left panels) and CV2 (α-chloroamide **2** binding, right panels) for all eight replicas across three independent bias-exchange metadynamics simulations. Each row represents an independent simulation run, and each horizontal strip within a panel corresponds to one replica trajectory. The color scale represents the CV values, with yellow indicating unbound states and blue indicating bound states. Multiple transitions between bound and unbound states are observed across all simulations, demonstrating extensive sampling of the configurational space. Trajectories were demultiplexed to recover continuous physical trajectories, ensuring observed transitions represent physical binding/unbinding events rather than replica exchange transitions.

S2.3. Loop flexibility analysis

To assess protein flexibility, root-mean-square fluctuation (RMSF) was calculated for the C_α atom of each residue, averaged over three independent simulation runs.

*Gk*OYE-G7 exhibits moderate flexibility with a mean RMSF of 2.26 ± 1.02 Å across all residues. To identify highly flexible regions, we applied a threshold of $\mu + 1.5\sigma$ (3.79 Å), where μ is the mean RMSF across all residues and σ is the standard deviation, highlighted as red bars in Figure S8. Four distinct regions exceeded this threshold: the N-terminal residue (M1), loop 3 (H105-M134), loop 5 (A218-L226), and loop 6 (S250-W264). Loop 6 exhibits the highest flexibility overall, and is expected to play a critical role in substrate binding. Interestingly, the mutation Y267W is located within this highly dynamic loop region. The calculated RMSFs correlate well with experimental B-factors[9], with the highest values observed for M1 (N-terminus), R257 (loop 6), and D183 (loop 3), confirming the dynamic nature of these regions.

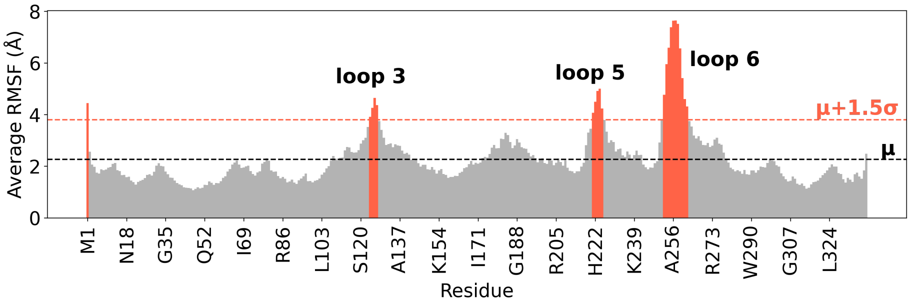

Figure S8. Average RMSF per residue calculated for C_α atoms ($n = 3$ independent runs). μ is the mean RMSF over all residues and σ the standard deviation. Red bars indicate residues exceeding the $\mu + 1.5\sigma$ threshold.

S2.4. Filtering MD trajectories to obtain catalytic poses

To identify catalytically relevant binding configurations from the extensive conformational sampling, we developed a filtering protocol that selects only those trajectory frames where both the substrates are positioned near FMN in geometries suitable for alkylation. This filtering is necessary because the bias-exchange metadynamics explores the entire binding landscape (Fig. 2a). The algorithm applies three sequential filters: (1) substrate binding (both substrates near the active site), (2) proximity requirements (substrates and FMN in close contact), and (3) reactive orientation (appropriate dihedral angle shown in Fig. S9).

Algorithm 1 Filtering MD trajectory frames to catalytically competent poses

```
for each frame in trajectory do
    {Step 1: Filter bound poses }
    if CV1 < 30.0 Å AND CV2 < 30.0 Å then
        {Step 2: Filter active site positioning to ensure substrates and FMN are in close contact}
        if distance(1, 2) < 10 Å AND distance(1, FMN) < 10 Å AND distance(2, FMN) < 10 Å then
            {Step 3: Filter reactive orientation}
            if θ ∈ [60°, 120°] OR [−60°, −120°] then
                Retain frame as catalytically competent
            end if
        end if
    end if
end for
```

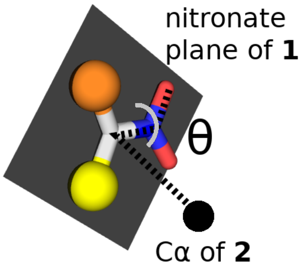

Figure S9. Dihedral angle θ definition. The $60° - 120°$ range avoids steric clashes with nitronate substituents (yellow and orange spheres) and orients the substrates for the reaction.

S2.5. Binding free energy of the α-chloroamide 2

α-chloroamide **2** shows lower binding affinity compared to nitronate **1** to *Gk*OYE-G7, with $\Delta G^{\circ}_{\text{bind}} = 0.5 \pm 0.3$ kcal/mol (Fig˙ S12). This weak affinity reflects amide's high solubility and lack of preferential binding to the protein over aqueous environment. As a tertiary amide, **2** can only engage in hydrogen-bonding interactions through its carbonyl group, which are equally stabilized in water as in the protein active site.

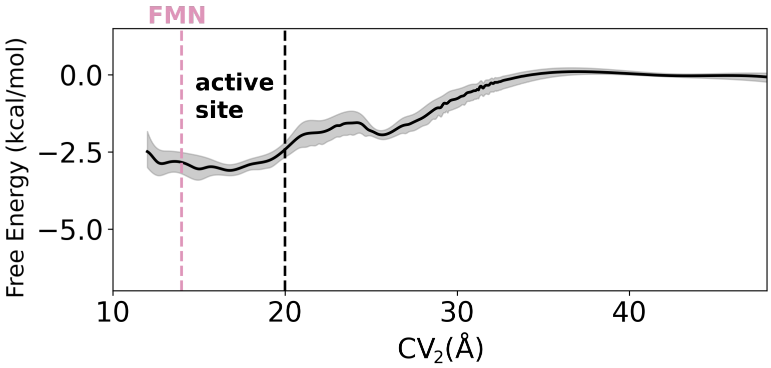

Figure S10. Calculated free energy profile for α-chloroamide **2** binding in *Gk*OYE-G7. CV2 represents the binding collective variable (Section S1.1, with FMN and active site positions indicated.

S2.6. Probing Y28 protonation state with constant pH MD simulations

To validate our choice of protonation state for Y28 at pH 9.0, we performed constant pH molecular dynamics simulations that allow protonation states to fluctuate. These simulations validated our assigned protonation state, confirming Y28 remains protonated and is a hydrogen-bond donor (Fig. S11).

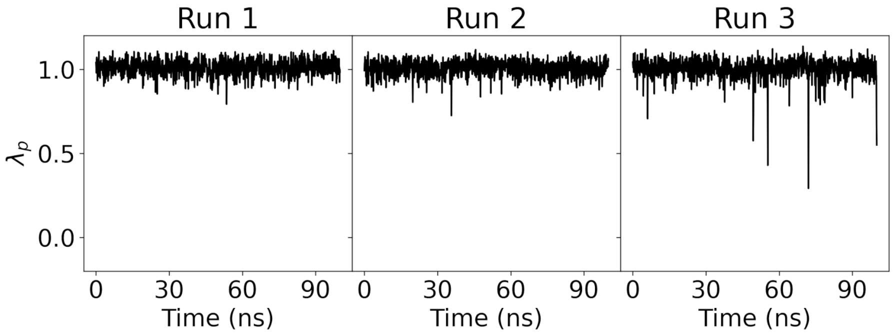

Figure S11. Constant pH MD validation of Y28 protonation state in catalytically competent geometries. Time evolution of Y28 protonation state over 100 ns simulations at pH 9.0 for three independent runs. $\lambda_p > 0.5$ indicates protonated tyrosine (hydrogen bond donor), $\lambda_p < 0.5$ indicates deprotonated tyrosine.

S2.7. QM/MM validation

Choice of the electronic structure method

QM/MM validation involves selecting an appropriate QM method that accurately describes reaction energies upon excitation (light absorption) and throughout the reaction coordinate (geometric changes). DFT is commonly employed in QM/MM calculations due to its favorable balance of computational cost and accuracy [10]. To assess whether DFT would be suitable for the C-alkylation/XEC reactions, we compared the DFT functional ωB97X-D [11] with multireference DCD-CAS(2) [12], a more computationally expensive but more accurate method for radical photochemistry.

For the overall reaction energy (energy difference between reactant and product), DCD-CAS(2) yielded -0.63 eV while ωB97X-D gave -0.85 eV. For cyan light excitation energies, DCD-CAS(2) and TD-DFT(ωB97X-D) showed excellent agreement (2.39 eV vs 2.41 eV, respectively). However, these properties primarily involve closed-shell geometries **I** and **IV**, either directly for the energy difference calculation or as reference ground-state electron densities for the TD-DFT linear response treatment.

Critical limitations of DFT emerged for the diradical geometry **II**. The DFT ground-state SCF solution was unstable, with stability analysis revealing a negative eigenvalue (-0.1515 Eh) in the electronic Hessian, indicating convergence to a saddle point rather than a true minimum. Although a stable broken-symmetry solution could be obtained, it exhibited significant spin contamination ($\langle S^2 \rangle = 1.03$ instead of 0.0), demonstrating substantial diradical character that violates the single-reference assumption underlying TD-DFT. Consequently, while TD-DFT performs adequately for equilibrium geometries with minimal diradical character, it fails ar certain geometries along the reaction path where multireference character becomes significant. Hence, we employed multireference CASSCF+DCD-CAS(2) methods for single-point energy calculations.

Geometry optimization validation

For geometry optimizations with NEB [13], we used finite temperature DFT [14,15] with PBEh-3c [16] functional to address the SCF convergence failure at diradical geometries. Figure S12 shows the evolution of electronic structure descriptors along the representative NEB pathway of Fig. 3a, with Hirshfeld charges [17] and HOMO-LUMO gap analysis. FMN and substrates begin with charges near -1, and the diradical region shows clear charge-transfer state formation (increased FMN charge, decreased substrate charge) followed by electron return to FMN, consistent with the proposed mechanism in Figures 3a and S14. The smooth HOMO-LUMO gap evolution through the diradical region (gap$\approx$ 0) demonstrates that finite temperature DFT converges where conventional DFT would fail. These qualitative trends indicate that ground state optimization with finite temperature DFT provides reasonable approximations to the diradical intermediate, supporting the applicability of this approach for reaction pathway determination.

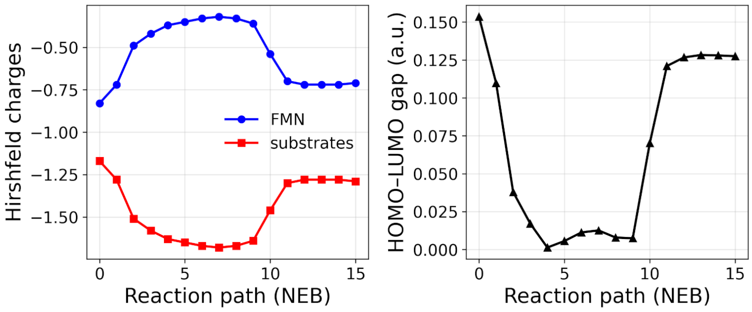

Figure S12. Finite temperature DFT properties along reaction path. (Left) Hirshfeld charges on FMN and substrates, showing charge-transfer state formation in the diradical region (increase FMN charge, decreased substrate charge). (Right) HOMO-LUMO gap evolution demonstrating the diradical region (gap ≈ 0) where conventional DFT methods would fail.

QM region size convergence

We systematically evaluated QM/MM convergence with respect to QM region size. The minimal QM region contains only the essential atoms required to describe light absorption and the reaction (see Methods in main text and Fig. S13). Larger QM regions were constructed by including additional atoms within spherical shells surrounding the substrates, and excitation energies were computed. Due to the unfavorable scaling of DCD-CAS(2), convergence testing was performed using ωB97X-D with the def2-SVP basis set. This approximation is justified since both methods yield very similar results for this geometry (Table S11). The convergence analysis assumes that the largest QM region (r_{QM} = 9 Å) is well-converged, since it includes substantial long-range electrostatic interactions. Interestingly, the excitation energy shows minimal dependence on QM region size, with the minimal region (2.40 eV) differing by only 0.04 eV from the largest region (2.44 eV). The intermediate r_{QM} = 6 Å region shows a slightly larger deviation (2.23 eV), suggesting possible error cancellation effects. Despite that, all tested QM regions consistently predict cyan/blue light excitation. Given the similar accuracy across all regions and the computational intractability of large QM regions with multireference methods, we employed the minimal QM region for our QM/MM calculations.

Table S11. Convergence of QM/MM excitation energy of pre-activated geometry **I** with increasing QM size

QM size	N. of atoms	Method	Excitation Energy (eV)
Minimal	68	DCD-CAS(2)/def2-TZVPP	2.39
Minimal	68	ωB97X-D/def2-TZVPP	2.41
Minimal	68	ωB97X-D/def2-SVP	2.40
r_{QM} = 6 Å	137	ωB97X-D/def2-SVP	2.23
r_{QM} = 9 Å	389	ωB97X-D/def2-SVP	2.44

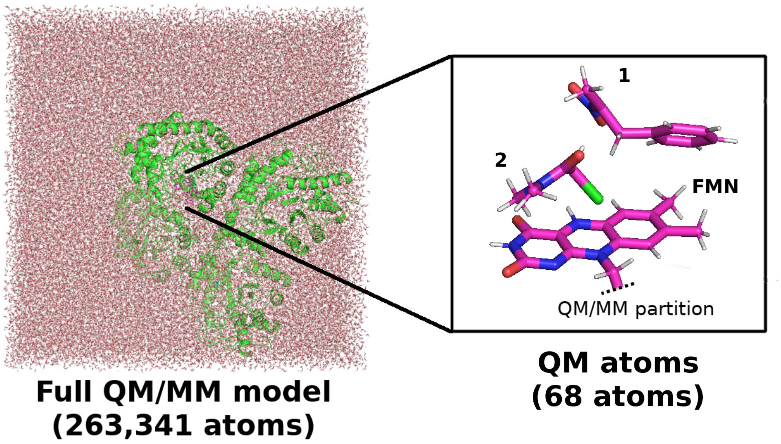

Figure S13. QM/MM model illustrating the complete all-atom system and the subset of atoms treated at the QM level (minimal region).

S2.8. Individual reaction paths

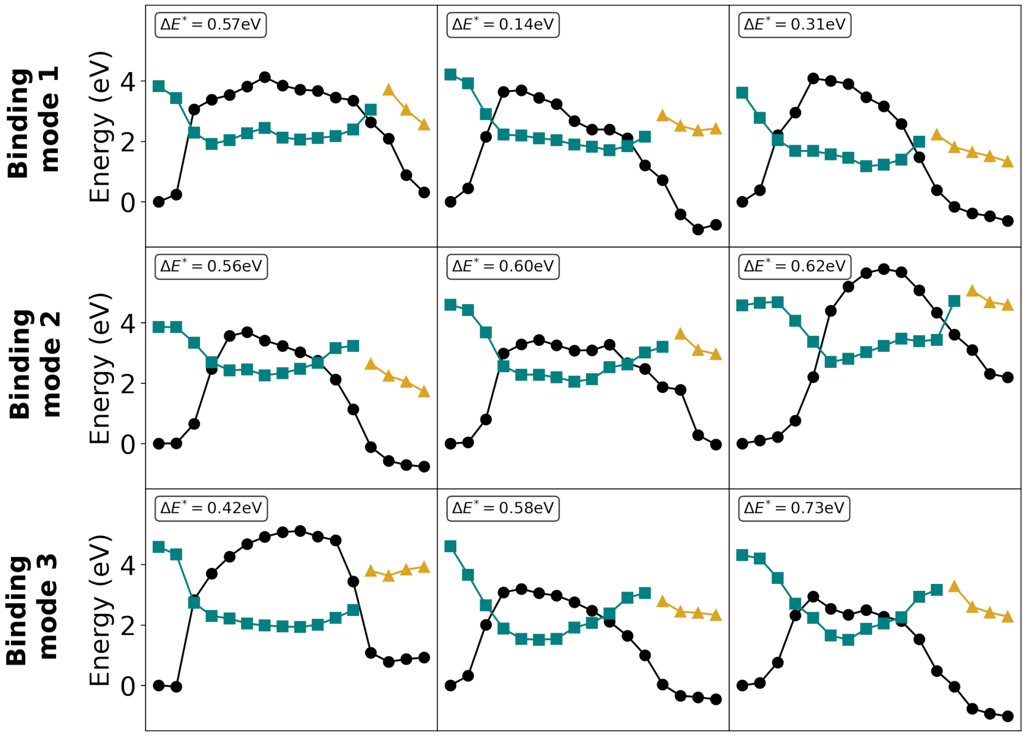

Figure S14. QM/MM-calculated reaction pathways determined using NEB optimizations for three randomly sampled configurations ($n = 3$) of binding modes 1 (top), 2 (middle), and 3 (bottom). SA[4]-CASSCF(4,4)/def2-TZVPP + DCDCAS(2) was used as QM method. Energy profiles show closed-shell electronic state (black), charge-transfer state (teal), and nitro-radical anion (orange). Activation barriers (ΔE^*) are calculated from the CT state minimum to the second crossing point, indicating binding mode 1 provides the most favorable reaction kinetics.

S2.9. Substrate steric and electronic descriptors and volume calculations

Binding pocket volumes were calculated in catalytic poses using the KVFinder webserver with default parameters. Solvent molecules, α-chloroamide **2** and the phenyl group of nitronate **1** were removed before conducting the analysis. To make the calculation faster, only cavities near FMN were searched. Substrate molecular volumes were computed using the RDKit Python library, which calculates van der Waals-type volumes from atomic radii and accounts for atomic overlap effects when provided with xyz coordinates from the optimized geometries.

Table S12. Steric and electronic descriptors of each α-halocarbonyl compound tested

Substrate	vdW volume (Å^3)	f_+	Bond dissociation energy (kcal/mol)
**2**	110.9	0.248	80.1
**3**	120.5	0.251	87.4
**4**	119.7	0.247	66.1
**5**	94.4	0.258	85.4
**6**	134.8	0.245	83.9
**7**	142.2	0.248	85.9
**8**	127.9	0.218	61.9
**9**	186.1	0.247	82.0
**10**	169.3	0.258	85.8
**11**	90.1	0.283	94.2
**12**	107.4	0.283	93.9

S3. Supporting References

[1] V. Limongelli, M. Bonomi, M. Parrinello, Funnel metadynamics as accurate binding free-energy method, *Proc. Natl. Acad. Sci.* **2013**, *110*, 6358. DOI: 10.1073/pnas.1303186110.

[2] F. Weigend, R. Ahlrichs, Balanced basis sets of split valence, triple zeta valence and quadruple zeta valence quality for H to Rn: Design and assessment of accuracy, *Phys. Chem. Chem. Phys.* **2005**, *7*, 3297. DOI: 10.1039/B508541A.

[3] J.-D. Chai, M. Head-Gordon, Systematic optimization of long-range corrected hybrid density functionals, *J. Chem. Phys.* **2008**, *128*, 084106. DOI: 10.1063/1.2834918.

[4] M. P. Kabir, P. Ghosh, S. Gozem, Electronic Structure Methods for Simulating Flavin's Spectroscopy and Photophysics: Comparison of Multi-reference, TD-DFT, and Single-Reference Wave Function Methods, *J. Phys. Chem. B* **2024**, *128*, 7545. DOI: 10.1021/acs.jpcb.4c03748.

[5] K. Vanommeslaeghe, E. Hatcher, C. Acharya, S. Kundu, S. Zhong, J. Shim, E. Darian, O. Guvench, P. Lopes, I. Vorobyov, A. D. Mackerell, Jr., CHARMM General Force Field: A Force Field for Drug-Like Molecules Compatible with the CHARMM All-Atom Additive Biological Force Fields, *J. Comput. Chem.* **2010**, *31*, 671. DOI: 10.1002/jcc.21367.

[6] W. L. Jorgensen, J. Chandrasekhar, J. D. Madura, R. W. Impey, M. L. Klein, Comparison of simple potential functions for simulating liquid water, *J. Chem. Phys.* **1983**, *79*, 926. DOI: 10.1063/1.445869.

[7] A. D. MacKerell, Jr., D. Bashford, M. Bellott, R. L. Dunbrack, Jr., J. D. Evanseck, M. J. Field, S. Fischer, J. Gao, H. Guo, S. Ha, D. Joseph-McCarthy, L. Kuchnir, K. Kuczera, F. T. K. Lau, C. Mattos, S. Michnick, T. Ngo, D. T. Nguyen, B. Prodhom, W. E. Reiher, B. Roux, M. Schlenkrich, J. C. Smith, R. Stote, J. Straub, M. Watanabe, J. Wiórkiewicz-Kuczera, D. Yin, M. Karplus, All-Atom Empirical Potential for Molecular Modeling and Dynamics Studies of Proteins, *J. Phys. Chem. B* **1998**, *102*, 3586. DOI: 10.1021/jp973084f.

[8] R. Leardi, Genetic algorithms in chemometrics and chemistry: a review, *J. Chemom.* **2001**, *15*, 559. DOI: 10.1002/cem.651.

[9] M. Schittmayer, A. Glieder, M. K. Uhl, A. Winkler, S. Zach, J. H. Schrittwieser, W. Kroutil, P. Macheroux, K. Gruber, S. Kambourakis, J. D. Rozzell, M. Winkler, Old Yellow Enzyme-Catalyzed Dehydrogenation of Saturated Ketones, *Adv. Synth. Catal.* **2011**, *353*, 268. DOI: 10.1002/adsc.201000862.

[10] H. M. Senn, W. Thiel, QM/MM Methods for Biomolecular Systems, *Angew. Chem. Int. Ed.* **2009**, *48*, 1198. DOI: 10.1002/anie.200802019.

[11] J.-D. Chai, M. Head-Gordon, Long-range corrected hybrid density functionals with damped atom–atom dispersion corrections, *Phys. Chem. Chem. Phys.* **2008**, *10*, 6615. DOI: 10.1039/B810189B.

[12] S. Pathak, L. Lang, F. Neese, A dynamic correlation dressed complete active space method: Theory, implementation, and preliminary applications, *J. Chem. Phys.* **2017**, *147*, 234109. DOI: 10.1063/1.5017942.

[13] V. Ásgeirsson, B. O. Birgisson, R. Bjornsson, U. Becker, F. Neese, C. Riplinger, H. Jṕnsson, Nudged Elastic Band Method for Molecular Reactions Using Energy-Weighted Springs Combined with Eigenvector Following, *J. Chem. Theory Comput.* **2021**, *17*, 4929. DOI: 10.1021/acs.jctc.1c00462.

[14] J.-D. Chai, Density functional theory with fractional orbital occupations, *J. Chem. Phys.* **2012**, *136*, 154104. DOI: 10.1063/1.3703894.

[15] A. D. Rabuck, G. E. Scuseria, Improving self-consistent field convergence by varying occupation numbers, *J. Chem. Phys.* **1999**, *110*, 695. DOI: 10.1063/1.478177.

[16] S. Grimme, J. G. Brandenburg, C. Bannwarth, A. Hansen, Consistent structures and interactions by density functional theory with small atomic orbital basis sets, *J. Chem. Phys.* **2015**, *143*, 054107. DOI: 10.1063/1.4927476.

[17] F. L. Hirshfeld, Bonded-Atom Fragments for Describing Molecular Charge Densities, *Theor. Chim. Acta* **1977**, *44*, 129. DOI: 10.1007/BF00549096.
